# PMN-MDSCs modulated by CCL20 from cancer cells promoted breast cancer cell stemness through CXCL2-CXCR2 pathway

**DOI:** 10.1038/s41392-023-01337-3

**Published:** 2023-03-01

**Authors:** Rui Zhang, Mengxue Dong, Juchuanli Tu, Fengkai Li, Qiaodan Deng, Jiahui Xu, Xueyan He, Jiajun Ding, Jie Xia, Dandan Sheng, Zhaoxia Chang, Wei Ma, Haonan Dong, Yi Zhang, Lixing Zhang, Lu Zhang, Suling Liu

**Affiliations:** 1 Fudan University Shanghai Cancer Center & Institutes of Biomedical Sciences; State Key Laboratory of Genetic Engineering; Cancer Institutes; Key Laboratory of Breast Cancer in Shanghai; The Shanghai Key Laboratory of Medical Epigenetics; Shanghai Key Laboratory of Radiation Oncology; The International Co-laboratory of Medical Epigenetics and Metabolism, Ministry of Science and Technology; Shanghai Medical College; Fudan University, Shanghai, 200032 China; 2 Breast Surgery, Obstetrics and Gynecology Hospital of Fudan University, Shanghai, China; 3 Department of Breast and Thyroid Surgery, Southwest Hospital, the First Affiliated Hospital of the Army Military Medical University, Chongqing, 400038 China; 4 Jiangsu Key Lab of Cancer Biomarkers, Prevention and Treatment, Collaborative Innovation Center for Cancer Medicine, Nanjing Medical University, Nanjing, 211166 China

**Keywords:** Cancer stem cells, Tumour immunology

## Abstract

Our previous studies have showed that C-C motif chemokine ligand 20 (CCL20) advanced tumor progression and enhanced the chemoresistance of cancer cells by positively regulating breast cancer stem cell (BCSC) self-renewal. However, it is unclear whether CCL20 affects breast cancer progression by remodeling the tumor microenvironment (TME). Here, we observed that polymorphonuclear myeloid-derived suppressor cells (PMN-MDSCs) were remarkably enriched in TME of CCL20-overexpressing cancer cell orthotopic allograft tumors. Mechanistically, CCL20 activated the differentiation of granulocyte-monocyte progenitors (GMPs) via its receptor C-C motif chemokine receptor 6 (CCR6) leading to the PMN-MDSC expansion. PMN-MDSCs from CCL20-overexpressing cell orthotopic allograft tumors (CCL20-modulated PMN-MDSCs) secreted amounts of C-X-C motif chemokine ligand 2 (CXCL2) and increased ALDH^+^ BCSCs via activating CXCR2/NOTCH1/HEY1 signaling pathway. Furthermore, C-X-C motif chemokine receptor 2 (CXCR2) antagonist SB225002 enhanced the docetaxel (DTX) effects on tumor growth by decreasing BCSCs in CCL20^high^-expressing tumors. These findings elucidated how CCL20 modulated the TME to promote cancer development, indicating a new therapeutic strategy by interfering with the interaction between PMN-MDSCs and BCSCs in breast cancer, especially in CCL20^high^-expressing breast cancer.

## Introduction

In terms of cancer-related deaths among women, breast cancer is the most prevalent.^1,2^ A small portion of malignant breast cancer cells with enhanced capacities for self-renewal and differentiation has been reported to contribute to therapy resistance, tumor recurrence and metastasis and named as breast cancer stem cells (BCSCs).^3–5^ Recent studies indicated that the stemness of cancer cells was not exclusively regulated by intrinsic signals. The interplay between cancer stem cells (CSCs) and TME has been extensively studied.^6–8^ It has been found that immune cells, such as tumor-associated macrophage (TAM), myeloid-derived suppressor cell (MDSC), regulatory T (Treg) cell, and dendritic cell (DC) interacted with CSCs through positive feedback loops.^9–12^ Several immune cells have been reported to trigger signaling pathway activation associated with CSCs by producing cytokines, such as interleukin 6 (IL6), tumor necrosis factor-α (TNF-α) and transforming growth factor-β (TGF-β).^13–15^ However, the factors that influence the self-renewal of BCSCs remain ambiguous.

Immature MDSCs with potent immunosuppressive activity accumulate during cancerous development.^16^ In mouse tumor model, two main MDSC subtypes have been characterized: monocytic myeloid-derived suppressor cells (M-MDSCs) labelled as CD11b^+^Ly6G^−^Ly6C^high^, and granulocytic polymorphonuclear MDSCs (PMN-MDSCs) labelled as CD11b^+^Ly6G^+^Ly6C^low^. M-MDSCs exhibit a stronger immunosuppressive effect compared to PMN-MDSCs.^16^ Nonetheless, PMN-MDSCs play an important role in the regulation of tumor featured immune responses.^17,18^ In cancer, MDSCs are significantly expanded and activated since they originate from myeloid progenitors.^19^ GMP differentiation into granulocyte progenitors (GPs) or monocyte progenitors (MPs) results in the production of PMN-MDSCs or M-MDSCs respectively.^20^

MDSCs have been reported to promote the cancer progression through supporting cell survival, invasion and metastases, and angiogenesis.^16,17,21^ Recently, MDSCs were identified as a modulator of CSCs. MDSCs elevated microRNA-101 expression in ovarian cancer cells and promoted the CSC phenotype.^22^ MDSCs were also reported to endow multiple myeloma cell stemness by inducing piRNA-823 expression and DNA methyltransferase 3 beta (DNMT3B) activation.^23^ Peng et al found that MDSCs promoted breast cancer cell stemness through activating IL-6/STAT3 and NO/NOTCH cross-talk signaling.^13^ PMN-MDSC-derived exosomal S100 calcium-binding protein A9 (S100A9) was reported to promote the stemness in colorectal cancer.^24^ However, the regulation and mechanism of PMN-MDSCs on BCSCs were unclear.

Usually CCL20 expression is low, but elevated during inflammation.^25–27^ Emerging evidence strongly suggested CCL20 was prominently upregulated in all breast cancer subtypes, especially basal-like subtypes.^28^ In agreement with previous reports, we recently identified that elevated level of CCL20 in blood serum and tumor tissues of breast cancer patients was linked to tumor malignancy and chemotherapy resistance.^28^ Some evidences showed that CCL20 might promote immunosuppressive TME to support tumor progression.^25,29^ For example, Tregs were induced and recruited to tumor tissues by CCL20 and increased colorectal cancer progression.^30,31^ High infiltration of CCR6^+^ Tregs suppressed the functions of IFNγ^+^CD8^+^ T cells to promote immunosuppression and disease progression in breast cancer.^32^ CCL20 also facilitated the recruitment of T helper type 17 (Th17) cells through upregulating IL6/CCAAT/enhancer-binding protein β in cervical cancer progression.^33^ Additionally, CCL20^high^ expression increased DC infiltration into breast cancer tissues and promoted immunosuppression by reprograming DCs.^34^ These results drove us to further elucidate the mechanism that CCL20 regulated immunosuppression in breast cancer progression.

Here, our study described that CCL20 promoted the differentiation of GMPs into GPs in the bone marrow (BM) by binding to its receptor CCR6, which resulted in a significant accumulation of PMN-MDSCs. CCL20-modulated PMN-MDSCs secreted amounts of CXCL2 and activated NOTCH1/HEY1 signaling pathway in breast cancer cells by binding to CXCR2, leading to the increase of ALDH^+^ BCSCs. CXCR2 knockdown in breast cancer cells diminished the PMN-MDSC-induced enhancement of breast cancer cell stemness. Furthermore, CXCR2 antagonist SB225002 combined with DTX in vivo not only dramatically inhibited the tumor growth but also significantly decreased stemness of breast cancer cells, suggesting CXCR2 may be a potential therapeutic target for breast cancer patients with high expression of CCL20.

## Results

### PMN-MDSC expansion was positively correlated with high expression of CCL20 in breast cancer

**Fig. 1:**
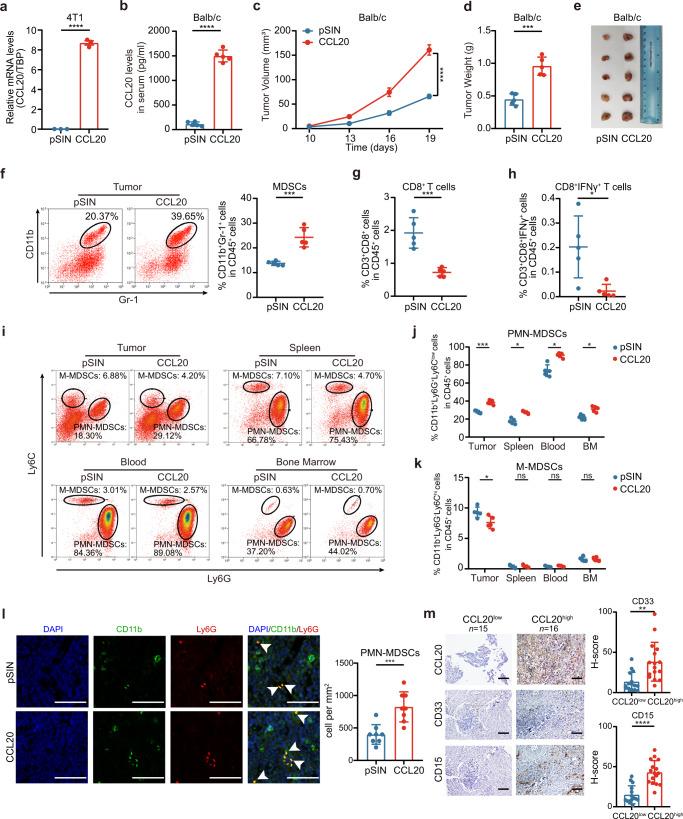
PMN-MDSCs were significantly augmented in mice bearing CCL20-overexpressing breast cancer cell orthotopic allograft tumors. **a** pSIN-/CCL20-overexpressing 4T1 cell lines were established, and overexpression efficiency of CCL20 was confirmed by qRT-PCR. Bar graph was presented as the mean of three biological independent experiments (mean ± SEM). Balb/c mice were orthotopically transplanted with pSIN-/CCL20-overexpressing 4T1 cells (5 × 10^4^) at the fourth mammary fat pads (*n* = 5 for each group). **b** The level of CCL20 in the blood serum of Balb/c mice was measured by ELISA and shown in bar graph as mean ± SEM. **c** Tumor size was monitored every 3 days, and tumor volume was calculated. **d, e** Tumor weight (**d**) and tumor image (**e**) was shown after the mice were sacrificed. **f** The percentage of MDSC (CD45^+^CD11b^+^Gr-1^+^) in pSIN-/CCL20-overexpressing 4T1 allograft tumors was analyzed by flow cytometry and shown in bar graph as mean ± SEM. **g**, **h** The percentage of CD3^+^CD8^+^ T cells (**g**) and the percentage of CD3^+^CD8^+^IFNγ^+^ T cells (**h**) from CD45^+^ cells in pSIN-/CCL20-overexpressing 4T1 cell allograft tumors were analyzed by flow cytometry and shown in bar graph as mean ± SEM. **i–k** The gating strategies for the analysis of PMN-MDSCs (CD45^+^CD11b^+^Ly6C^low^Ly6G^+^) and M-MDSCs (CD45^+^CD11b^+^Ly6C^high^Ly6G^−^) by flow cytometry were shown (**i**). The percentage of PMN-MDSC (**j**) and M-MDSC (**k**) in tumor, spleen, blood, and BM of Balb/c mice bearing pSIN-/CCL20-overexpressing 4T1 cell allograft tumors were shown in bar graph as mean ± SEM. **l** The percentage of CD11b-positive cells and Ly6G-positive cells in Balb/c mice bearing pSIN-/CCL20-overexpressing 4T1 cell allograft tumors were analyzed by IF staining. Scale bar, 50 μm. **m** CD33 and CD15 expression in CCL20^high^ and CCL20^low^ tumor tissues from breast cancer patients were analyzed by IHC staining. Representative images and the graph of H Scores (mean ± SEM) were shown. Scale bar, 100 μm. ns, no significance; *p < 0.05, **p < 0.01, ***p < 0.001, ****p < 0.0001

To investigate whether CCL20 promoted cancer progression via remodeling TME, we established tumor-bearing Balb/c and C57BL/6N mouse models, which were orthotopically transplanted with pSIN-/CCL20-overexpressing 4T1 and Py8119 cells, respectively. CCL20 overexpression was confirmed at mRNA level by qRT-PCR and at the protein level in the blood serum of mice bearing CCL20-overexpressing breast cancer cell orthotopic allograft tumors by mouse CCL20 ELISA kit (Fig. 1a, b and Supplementary Fig. S1). We observed that CCL20 overexpression remarkably promoted tumor growth (Fig. 1c–e and Supplementary Fig. S2). Gene Expression Profiling Interactive Analysis 2 (GEPIA2) database showed that the expression of CCL20 was positively correlated with the expression of CD11b (a myeloid marker in human, *p* value = 6.8e-25, R = 0.31) in patients’ breast tumors (Supplementary Fig. S3).

In our study, in line with a substantial expansion of MDSCs (Fig. 1f), a dramatic decrease of CD8^+^ T cells, especially CD8^+^IFNγ^+^ T cells (Fig. 1g, h), was observed in CCL20-overexpressing 4T1 tumors. No change was observed in macrophages, including inflammation-promoting M1-like as well as anti-inflammatory and immunosuppressive M2-like macrophages, DCs, and natural killer (NK) cells (Supplementary Fig. S4). Consistent with the results in tumor mouse models, the expression of CCL20 was positively correlated with the expression of CD33 (a MDSC marker in human, *p* value = 1.2e-15, R = 0.24) but not CD68 (a pan-macrophage marker in human, *p* value = 0.011, R = 0.077) in patients’ breast tumors from GEPIA2 database (Supplementary Fig. S5), indicating that CCL20 overexpression facilitated cancer cells to create a tolerogenic environment.

To further determine which MDSC subtype was increased in CCL20-overexpressing cell allograft tumors, the proportions of PMN-MDSCs and M-MDSCs were analyzed based on cell surface marker expression. Results of flow cytometry and immunofluorenscence (IF) consistently showed that the PMN-MDSC percentage was significantly increased in CCL20-overexpressing cell allograft tumors (Fig. 1i, j, l and Supplementary Fig. S6a). Consistently, the PMN-MDSC percentage in the spleen, blood, and BM was also elevated (Fig. 1i, j and Supplementary Figs. S6a, S7). In contrast, the M-MDSC percentage was decreased, indicating PMN-MDSCs were the main contributor to the MDSC expansion in CCL20-overexpressing cell allograft tumors (Fig. 1k and Supplementary Fig. S6b). Furthermore, we examined the expressions of CD33 and CD15 in 31 breast tumor samples by immunohistochemistry (IHC) and found staining scores of these two PMN-MDSC markers were much higher in CCL20^high^-expressing tumors than in CCL20^low^-expressing tumors (Fig. 1m). These results confirmed that CCL20 overexpression in breast cancer cells promoted PMN-MDSC expansion, which might suppress host immunity activity.

### CCL20 promoted PMN-MDSC expansion by inducing GMP differentiation to GPs

**Fig. 2:**
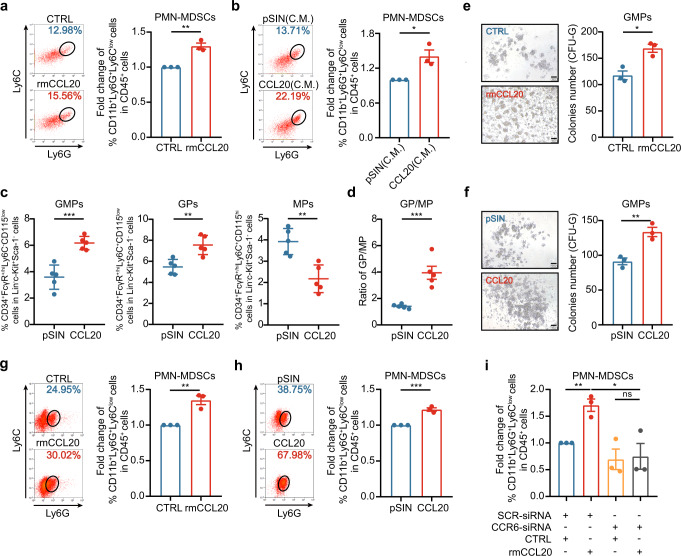
CCL20 stimulated the PMN-MDSC expansion by inducing GMP differentiation. **a**, **b** BM cells from mice bearing 4T1 cell allograft tumors were treated with rmCCL20 (10 ng/ml) (**a**) or conditioned medium (C.M.) of pSIN-/CCL20-overexpressing 4T1 cells (**b**) for 6 days. The PMN-MDSC percentage in BM was analyzed by flow cytometry and shown in bar graph as mean ± SEM. **c**, **d** Balb/c mice were orthotopically transplanted with pSIN-/CCL20-overexpressing 4T1 cells (5×10^4^) at the fourth mammary fat pads (*n* = 5 for each group). After mice were sacrificed, the percentage of bone marrow progenitor cells, including GMPs (Lin^−^c-Kit^+^Sca-1^−^CD34^+^FcγR^+/high^Ly6C^−^CD115^low^), GPs (Lin^−^c-Kit^+^Sca-1^−^CD34^+^FcγR^+/high^Ly6C^+^CD115^low^) and MPs (Lin^−^c-Kit^+^Sca-1^−^CD34^+^FcγR^+/high^Ly6C^+^CD115^high^) were analyzed by flow cytometry (**c**), and the ratio of GP/MP was shown as mean ± SEM (**d**). **e** GMPs were sorted from BM of mice bearing 4T1 cell allograft tumors and treated with rmCCL20 (10 ng/ml). One thousand GMPs per well were cultured in the methylcellulose-based medium for CFU assay for 10 days. The representative images of CFU-G were shown (left), and the number of CFU-G (right) was counted in bar graph as mean ± SEM. Scale bar, 100 μm. **f** GMPs were sorted from BM of mice bearing pSIN-/CCL20-overexpressing 4T1 cell allograft tumors. One thousand GMPs per well were cultured in the methylcellulose-based medium for CFU assay for 10 days. The representative images of CFU-G were shown (left), and the number of CFU-G (right) was counted in bar graph as mean ± SEM. Scale bar, 100 μm. **g**, **h** The cells from the GMP-derived colonies were collected, and the PMN-MDSC percentage was analyzed by flow cytometry and shown in bar graph as mean ± SEM. **i** GMPs were sorted from BM of mice bearing 4T1 cell allograft tumors. After being transiently transfected with CCR6-siRNA, GMPs were treated with rmCCL20 (10 ng/ml) or PBS for 10 days, and then the PMN-MDSC percentage was analyzed by flow cytometry. Bar graph was presented as the mean of three biological independent experiments (mean ± SEM). ns, no significance; *p < 0.05, **p < 0.01, ***p < 0.001

To investigate how CCL20 induced PMN-MDSC expansion in tumors, we first isolated BM cells from 4T1 allograft mouse model and then treated BM cells with recombination mouse CCL20 protein (rmCCL20). After three days, we observed that rmCCL20 significantly increased PMN-MDSC percentage in BM cells (Fig. 2a). Isolated BM cells were also cultured with conditioned medium of pSIN-/CCL20-overexpressing 4T1 cells. PMN-MDSC expansion was more pronounced in BM cells cultured with the conditioned medium of CCL20-overexpressing cells (Fig. 2b).

Based on the results in vitro, we speculated that CCL20 might regulate BM progenitor cell differentiation, contributing to PMN-MDSC expansion. GMP is the point of bifurcation between monocytic and granulocytic differentiation.^20^ We found the percentage of GMPs and GMP-derived GPs were significantly increased whereas MPs were decreased in BM of mice bearing CCL20-overexpressing 4T1 cell orthotopic allograft tumors by flow cytometry (Fig. 2c and Supplementary Fig. S8a, b). The elevated GP/MP ratio reinforced the expansion of GPs (Fig. 2d and Supplementary Fig. S8c), revealing that CCL20 skewed GMP differentiation toward the granulocytic lineage, which gave rise to PMN-MDSCs.

In methylcellulose cultures supplemented with hematopoietic cytokines, GMPs formed colony-forming units (CFUs) including granulocyte-monocyte colony (CFU-GM), granulocyte-colony (CFU-G) and monocyte-colony (CFU-M), but GPs only gave rise to CFU-G.^35^ To determine whether PMN-MDSC expansion was due to GMP differentiation by CCL20, we treated GMPs from BM of mice bearing 4T1 cell allograft tumors with rmCCL20 and observed that CFU-G, not CFU-M, was strikingly increased in the presence of rmCCL20 (Fig. 2e and Supplementary Fig. S8d, e). Consistently, GMPs sorted from BM of mice bearing CCL20-overexpressing 4T1 cell allograft tumors formed more CFU-G colonies (Fig. 2f and Supplementary Fig. S8f). We further collected the cells from the GMP-derived colonies and found that PMN-MDSC percentage in the colonies derived from GMPs treated with rmCCL20 or sorted from mice bearing CCL20-overexpressing 4T1 cell allograft tumors was significantly increased (Fig. 2g, h). Additionally, The CFU-G number and the PMN-MDSC percentage in CFU-G colonies derived from GPs treated with rmCCL20 or sorted from mice bearing CCL20-overexpressing 4T1 cell allograft tumors were not changed in comparison to controls (Supplementary Fig. S8g–j). These results confirmed that CCL20 induced the PMN-MDSC expansion via promoting the differentiation of GMPs to GPs.

To date, only CCR6 was reported as the CCL20 receptor.^36,37^ To verify whether CCL20 induced GMP differentiation through binding to CCR6, CCR6 was knocked down by siRNAs in GMPs (Supplementary Fig. S9a–d), and then CFU assay and flow cytometry were performed. The results showed that CCR6 knockdown suppressed CCL20-induced GMP differentiation (Fig. 2i and Supplementary Fig. S9e), suggesting that CCL20 promoted the differentiation of GMPs to GPs through CCR6.

### CCL20-modulated PMN-MDSCs enhanced the stemness of breast cancer cells

**Fig. 3:**
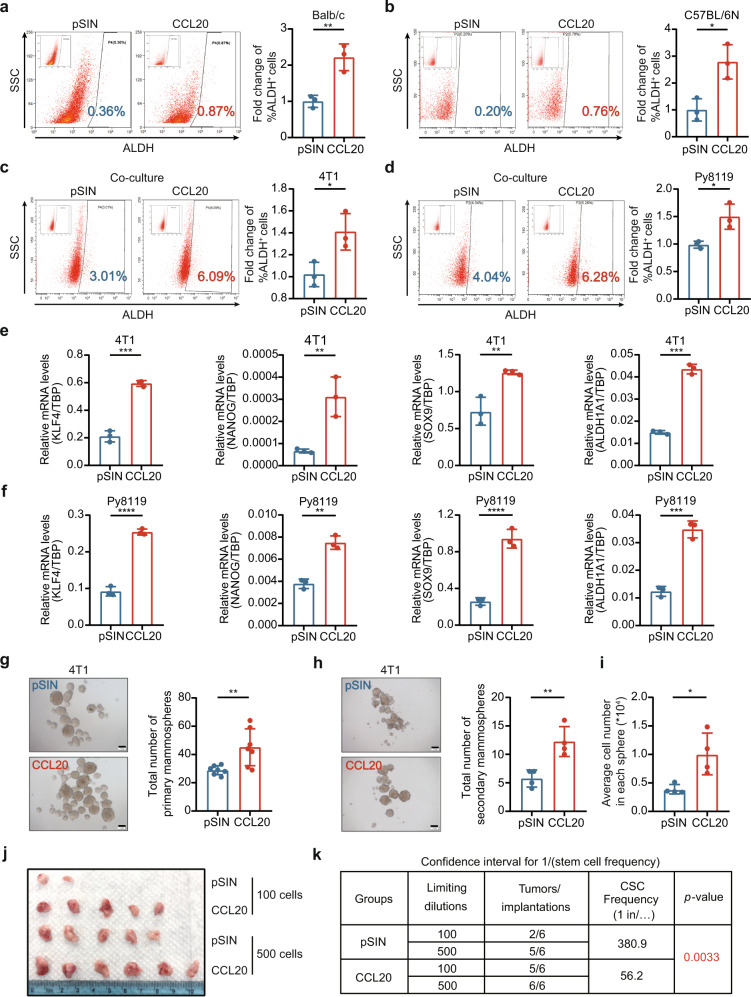
CCL20-modulated PMN-MDSCs enhanced the stemness of breast cancer cells. Balb/c mice or C57BL/6N mice were orthotopically transplanted with pSIN-/CCL20-overexpressing 4T1 or Py8119 cells at the fourth mammary fat pads, respectively. **a, b** The percentage of ALDH^+^ BCSCs was determined by ALDEFLUOR assay in tumor cells (CD45^−^CD140b^−^CD31^−^) from tumors of Balb/c mice (**a**) or C57BL/6N mice (**b**). Bar graph was presented as the mean of three biologically independent experiments (mean ± SEM). **c**, **d** PMN-MDSCs, sorted from pSIN-/CCL20-overexpressing 4T1 or Py8119 cell allograft tumors, were labeled as pSIN^PMN^ or CCL20^PMN^ in Figures and as pSIN-PMN-MDSCs or CCL20-modulated PMN-MDSCs in main text. 4T1 or Py8119 cells were co-cultured with pSIN-/CCL20-modulated PMN-MDSCs for 3 days. The percentage of ALDH^+^ BCSCs was determined by ALDEFLUOR assay in 4T1 (**c**) or Py8119 cells (**d**). Bar graph was presented as the mean of three biologically independent experiments (mean ± SEM). **e**, **f** 4T1 or Py8119 cells were co-cultured with pSIN-/CCL20-modulated PMN-MDSCs for 3 days. The mRNA expression levels of several stemness-related genes were analyzed in 4T1 (**e**) or Py8119 cells (**f**) by qRT-PCR. Bar graph was presented as the mean of three biologically independent experiments (mean ± SEM). **g**–**i** Self-renewal capability was determined by forming both primary mammospheres (**g**, *n* = 7 for each group) and secondary mammospheres (**h**, *n* = 4 for each group) in 4T1 cells mix-cultured with pSIN-/CCL20-modulated PMN-MDSCs. The average cell number of each secondary mammosphere was shown (**i**), and bar graph was presented as mean ± SEM. Scale bar, 1 mm. **j**, **k** 4T1 cells were mix-cultured with pSIN-/CCL20-modulated PMN-MDSCs for 3 days, and then sorted by flow cytometry and engrafted to the fourth mammary fat pads of Balb/c mice at a limited dilution (*n* = 3 for each group, two injection sites each mouse, 100 or 500 cells/site). Tumor image was shown (**j**). The stem cell frequency in tumor tissue was calculated by the limited dilution assay (**k**). The stem cell frequency and *p*-value calculation were based on the positive tumor sites. *p < 0.05, **p < 0.01, ***p < 0.001, ****p < 0.0001

Breast cancer progression and resistance to therapy are believed to be primarily caused by BCSCs.^38,39^ We observed that, besides the expansion of PMN-MDSCs, ALDH^+^ BCSCs, not CD24^+^CD29^+^ BCSCs,^5^ were increased significantly in CCL20-overexpressing tumors (Fig. 3a, b and Supplementary Fig. S10). To determine whether CCL20-modulated PMN-MDSCs played a critical role in promoting the stemness of breast cancer cells, pSIN- or CCL20-modulated PMN-MDSCs sorted from pSIN-/CCL20-overexpressing cell allograft tumors were co-cultured or mix-cultured with breast cancer cells for three days and the percentage of ALDH^+^ BCSCs was analyzed by flow cytometry. Results showed that CCL20-modulated PMN-MDSCs markedly elevated the percentage of ALDH^+^ BCSCs (Fig. 3c, d and Supplementary Fig. S11a). However, there was no change for the percentage of ALDH^+^ BCSCs in 4T1 cells co-cultured with M-MDSCs sorted from CCL20-overexpressing cell allograft tumors (Supplementary Fig. S11b). These results indicated that CCL20-modulated PMN-MDSCs induced the enrichment of ALDH^+^ BCSCs. In addition, the expressions of four stemness-related genes including *Klf4*, *Nanog*, *Sox9* and *Aldh1a1* were upregulated in 4T1 or Py8119 cells co-cultured with CCL20-modulated PMN-MDSCs (Fig. 3e, f). Furthermore, we assessed the in vitro self-renewal ability of 4T1 cells mix-cultured with pSIN- or CCL20-modulated PMN-MDSCs. As shown in Fig. 3g to i, CCL20-modulated PMN-MDSCs obviously promoted tumor sphere formation, and the mRNA expression levels of four stemness-related genes in primary mammosphere were remarkably increased (Supplementary Fig. S11c).

Next, the limited dilution assay (LDA) was used to investigate the effect of CCL20-modulated PMN-MDSCs on BCSC self-renewal in vivo. At first, 100 and 500 4T1 cells co-cultured with pSIN- or CCL20-modulated PMN-MDSCs were injected in the mammary fat pads of female mice. Then, tumor volume was monitored for one month, The results of in vivo LDA demonstrated that the frequency of tumor-initiating cells was increased approximately six-fold in the group of 4T1 cells co-cultured with CCL20-modulated PMN-MDSCs in comparison to the pSIN group (Fig. 3j, k). Collectively, these results indicated that CCL20-modulated PMN-MDSCs enhanced the stemness and self-renewal ability of breast cancer cells.

### CCL20-modulated PMN-MDSCs enhanced breast cancer cell stemness through the CXCL2-CXCR2 pathway

**Fig. 4:**
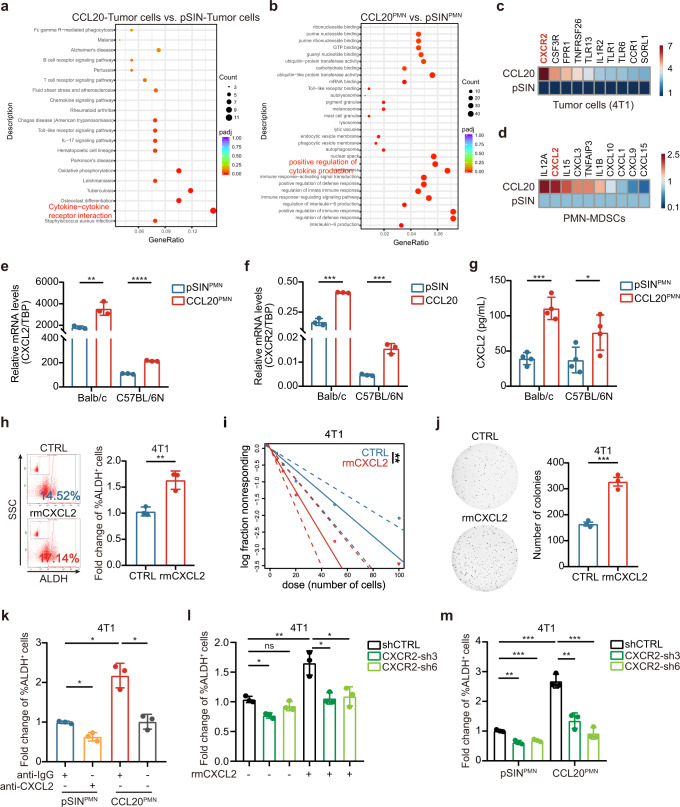
CXCL2 secreted by CCL20-modulated PMN-MDSCs enhanced the stemness through CXCR2. **a**–**d** Tumor cells and PMN-MDSCs were sorted from pSIN-/CCL20-overexpressing 4T1 cell allograft tumors. The total RNA of pSIN-/CCL20-modulated PMN-MDSCs (pSIN^PMN^/CCL20^PMN^) or tumor cells were extracted and used for RNA-Seq analysis. Bubble charts of gene function annotation and enrichment from tumor cells (**a**) or PMN-MDSCs (**b**) in pSIN-/CCL20-overexpressing 4T1 cell allograft tumors were shown. Significantly upregulated chemokines receptors in tumor cells (**c**) or chemokines from PMN-MDSCs (**d**) were screened and shown as heatmaps. The order was rearranged by variation based on the fold change. **e, f** qRT-PCR was performed to determine the mRNA expression levels of CXCL2 in pSIN^PMN^ or CCL20^PMN^ (**e**) and CXCR2 in tumor cells (**f**) sorted from pSIN-/CCL20-overexpressing 4T1 cell allograft tumors. Data were presented as mean ± SEM. **g** pSIN^PMN^ or CCL20^PMN^ from pSIN-/CCL20-overexpressing 4T1 or Py8119 cell allograft tumors were cultured for 2 days. The level of CXCL2 in the culture medium was measured and normalized by ELISA. Bar graph was shown as mean ± SEM. **h** 4T1 cells were treated with rmCXCL2 (10 ng/ml) for 3 days, and the percentage of ALDH^+^ BCSCs was determined by ALDEFLUOR assay in 4T1 cells. Bar graph was presented as the mean of three biologically independent experiments (mean ± SEM). **i** In vitro LDA of 4T1 cells treated with rmCXCL2 (10 ng/ml) for 3 days**. j** 4T1 cells were treated with rmCXCL2 (10 ng/ml) for 3 days, and then the soft agar colony formation assay was performed. After 4–6 weeks, the colony images were taken (left), and the colony numbers were quantified (right). Bar graph was presented as the mean of three biologically independent experiments (mean ± SEM). **k** 4T1 cells were co-cultured with pSIN^PMN^ or CCL20^PMN^ from 4T1 cell allograft tumors. Then, the CXCL2 neutralizing antibody (anti-CXCL2, 2 µg/ml) or control (anti-IgG, 2 µg/ml) was administrated for 3 days, and the percentage of ALDH^+^ BCSCs was determined by ALDEFLUOR assay in 4T1 cells. Bar graph was presented as the mean of three biologically independent experiments (mean ± SEM). **l** Cells were treated with rmCXCL2 (10 ng/ml) or PBS for 3 days. The percentage of ALDH^+^ BCSCs was determined by ALDEFLUOR assay in 4T1 cells, and bar graph was presented as mean ± SEM. **m** Cells were co-cultured with pSIN^PMN^ or CCL20^PMN^ sorted from 4T1 cell allograft tumors for 3 days. the percentage of ALDH^+^ BCSCs was determined by ALDEFLUOR assay in 4T1 cells, and bar graph was presented as the mean of three biologically independent experiments (mean ± SEM). ns, no significance; *p < 0.05, **p < 0.01, ***p < 0.001, ****p < 0.0001

To explore the mechanism that CCL20-modulated PMN-MDSCs promoted stemness of breast cancer cells, we sorted tumor cells and PMN-MDSCs from pSIN-/CCL20-overexpressing 4T1 cell allograft tumors and examined the transcriptome profiling via RNA-Seq. KEGG pathway gene set enrichment analysis (GSEA) revealed that chemokine signaling pathways were enriched in both tumor cells and PMN-MDSCs (Fig. 4a, b). We screened top ten upregulated genes in chemokine signaling pathways and the top fifty up-regulated genes in all sequenced genes, and consistently found CXCL2 and its receptor CXCR2 were significantly upregulated in CCL20-modulated PMN-MDSCs and tumor cells, respectively (Fig. 4c, d and Supplementary Fig. S12a, b). The upregulated mRNA levels of CXCL2 and CXCR2 were further confirmed by qRT-PCR (Fig. 4e, f). In addition, more CXCL2 was present in the conditioned medium of CCL20-modulated PMN-MDSCs compared to pSIN group as measured by mouse CXCL2 ELISA kit (Fig. 4g).

To verify whether CCL20-modulated PMN-MDSCs promoted stemness of breast cancer cells via CXCL2-CXCR2 axis, 4T1 or Py8119 cells were treated with recombination mouse CXCL2 protein (rmCXCL2) for three days. The results of flow cytometry (Fig. 4h and Supplementary Fig. S13a), qPCR (Supplementary Fig. S13b, c), in vitro LDA (Fig. 4i and Supplementary Fig. S13d), and soft agar anchorage-independent growth (AIG) assay (Fig. 4j) consistently showed that rmCXCL2 increased the stemness properties of breast cancer cells. The increase of ALDH^+^ BCSC percentage in 4T1 or Py8119 cells co-cultured with CCL20-modulated PMN-MDSCs was blocked by CXCL2 neutralizing antibody (Fig. 4k and Supplementary Fig. S14a). Furthermore, utilizing qRT-PCR (Supplementary Fig. S14b, c), in vitro LDA (Supplementary Fig. S14d, e), and AIG assay (Supplementary Fig. S14f), we also confirmed that CXCL2 neutralizing antibody inhibited breast cancer cell stemness promoted by CCL20-modulated PMN-MDSCs, suggesting CCL20-modulated PMN-MDSCs promoted the cancer cell stemness by secreting amounts of CXCL2.

We sequentially evaluated the protein expression of CXCR2 in ALDH^+^ BCSCs and found that CXCR2 protein was highly expressed in ALDH^+^ BCSCs by western blotting (Supplementary Fig. S15), suggesting that CXCR2 might play a critical role in activating the stemness of breast cancer. Thus, we treated scramble or CXCR2-knockdown 4T1 cell lines (Supplementary Fig. S16) with rmCXCL2 for three days. CXCR2 knockdown significantly inhibited the increase of ALDH^+^ BCSCs induced by rmCXCL2 (Fig. 4l). Similar results were obtained using a co-culture system (Fig. 4m). In addition, mammosphere formation ability and stem-related gene expression were consistently suppressed significantly in CXCR2-knockdown cells (Supplementary Fig. S17). These data indicated that CCL20-modulated PMN-MDSCs enhanced the stemness of BCSCs through the CXCL2-CXCR2 pathway.

### The CXCL2-CXCR2 axis enhanced the stemness of breast cancer cells relying on NOTCH1/HEY1

**Fig. 5:**
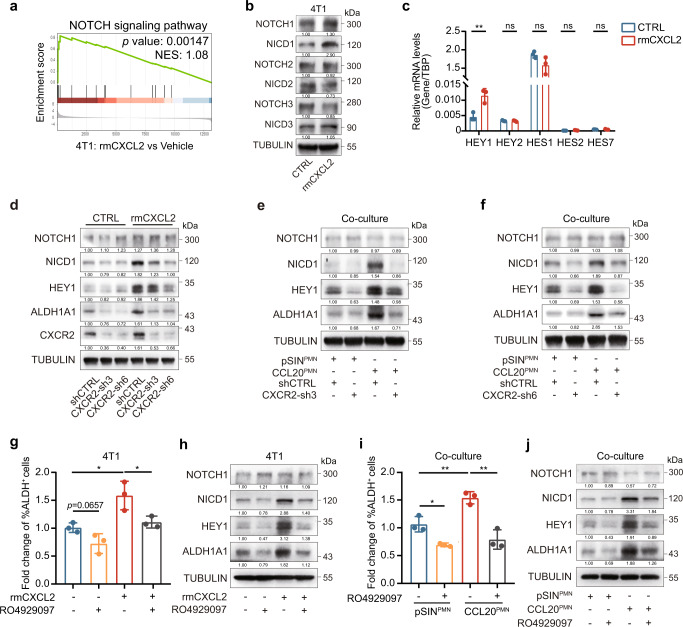
CXCL2-CXCR2 axis enhanced the stemness of breast cancer cells through activating NOTCH1/HEY1 pathway. **a** GSEA showed the enrichment for NOTCH-related genes with increased expression in 4T1 cells treated with rmCXCL2 (10 ng/ml) for 3 days. The *p* value was shown. **b** The protein expression levels of NOTCH1/2/3 and NICD1/2/3 in 4T1 cells treated with rmCXCL2 (10 ng/ml) or PBS for 3 days were analyzed by western blotting. **c** The mRNA expression levels of *HEY1*, *HEY2*, *HES1*, *HES2* and *HES7* in 4T1 cells treated with rmCXCL2 (10 ng/ml) or PBS for 3 days were analyzed by qRT-PCR. Bar graph was presented as the mean of three biologically independent experiments (mean ± SEM). **d** Cells were treated with rmCXCL2 (10 ng/ml) or PBS for 3 days, and the protein levels were analyzed by western blotting. **e, f** PMN-MDSCs were sorted from pSIN-/CCL20-overexpressing 4T1 cell allograft tumors (pSIN^PMN^/CCL20^PMN^). 4T1 scramble (shCTRL) or CXCR2-knockdown cells (CXCR2-sh3, **e**; CXCR2-sh6, **f**) were co-cultured with pSIN^PMN^ or CCL20^PMN^ for 3 days. The protein expression levels of NOTCH1, NICD1, HEY1, and ALDH1A1 in 4T1 cells were analyzed by western blotting. **g, h** 4T1 cells were treated with rmCXCL2 (10 ng/ml) or/and NOTCH inhibitor RO4929097 (1 µM) for 3 days. The percentage of ALDH^+^ BCSCs was determined by ALDEFLUOR assay in 4T1 cells, and bar graph was shown as mean ± SEM (**g**). The protein expression levels in 4T1 cells were analyzed by western blotting (**h**). **i, j** 4T1 cells were co-cultured with pSIN^PMN^ or CCL20^PMN^ and simultaneously treated with RO4929097 (1 µM) or DMSO for 3 days. The percentage of ALDH^+^ BCSCs was determined by ALDEFLUOR assay in 4T1 cells (**i**), and the protein levels of NOTCH1, NICD1, HEY1, and ALDH1A1 (**j**) in 4T1 cells were analyzed by western blotting. Bar graph was presented as the mean of three biologically independent experiments (mean ± SEM). ns, no significance; *p < 0.05, **p < 0.01

To reveal the signaling pathway involved in CXCL2-promoted breast cancer cell stemness, we treated 4T1 cells with rmCXCL2 or PBS and performed RNA-Seq analysis. Bioinformatic analysis revealed that CXCL2 activated the NOTCH signaling pathway, especially NOTCH1 signaling pathway (Fig. 5a and Supplementary Fig. S18). Previous studies have shown that NOTCH signaling is important for cancer progression and BCSC regulation.^40,41^ Hairy and enhancer of split (HES) as well as hes related family bHLH transcription factor with YRPW motif (HEY) families are both normally considered primary downstream targets of NOTCH signaling and contribute to cell fate determination.^42^ Therefore, the protein expressions of NOTCH1/2/3 and their intracellular domains NICD1/2/3 were determined by western blotting, and mRNA levels of downstream genes in HES and HEY families were analyzed by qRT-PCR. The results showed that NICD1 protein expression level and HEY1 mRNA level were remarkably upregulated (Fig. 5b, c and Supplementary Fig. S19a). However, the protein expressions of NICD1, HEY1, and Aldehyde dehydrogenase 1A1 (ALDH1A1) were inhibited significantly in CXCR2-knockdown 4T1 cell lines (Fig. 5d and Supplementary Fig. S19b). Consistent results were obtained using the co-culture system (Fig. 5e, f and Supplementary Fig. S19c, d). These results indicated that NOTCH1/HEY1 signaling pathway was involved in CXCL2-CXCR2 axis-induced enrichment of BCSCs.

To validate the role of NOTCH1/HEY1 pathway on mediating CXCL2-induced breast cancer cell stemness, 4T1 cells were treated with rmCXCL2 and NOTCH inhibitor RO4929097, a γ-secretase inhibitor to reduce NICD1 by blocking transmembrane proteolytic cleavage.^43^ The increase of ALDH^+^ BCSCs induced by rmCXCL2 was inhibited in 4T1 cells under RO4929097 treatment (Fig. 5g). The elevated expressions of NICD1, HEY1, ALDH1A1 by rmCXCL2 were also decreased in 4T1 cells after RO4929097 treatment (Fig. 5h and Supplementary Fig. S19e). Consistent with the results, RO4929097 treatment strikingly inhibited ALDH^+^ BCSCs and the protein expressions of NICD1, HEY1, ALDH1A1 in 4T1 cells co-cultured with CCL20-modulated PMN-MDSCs (Fig. 5i, j and Supplementary Fig. S19f). Consistent results were observed in Py8119 cells (Supplementary Figs. S20, S21). Taken together, we confirmed that CXCL2-CXCR2 axis, which was activated by CCL20-modulated PMN-MDSCs, enhanced the stemness of breast cancer cells by NOTCH1/HEY1 signaling pathway.

### CXCR2 antagonist enhanced the therapeutic efficacy of DTX on CCL20-expressing breast tumors

**Fig. 6:**
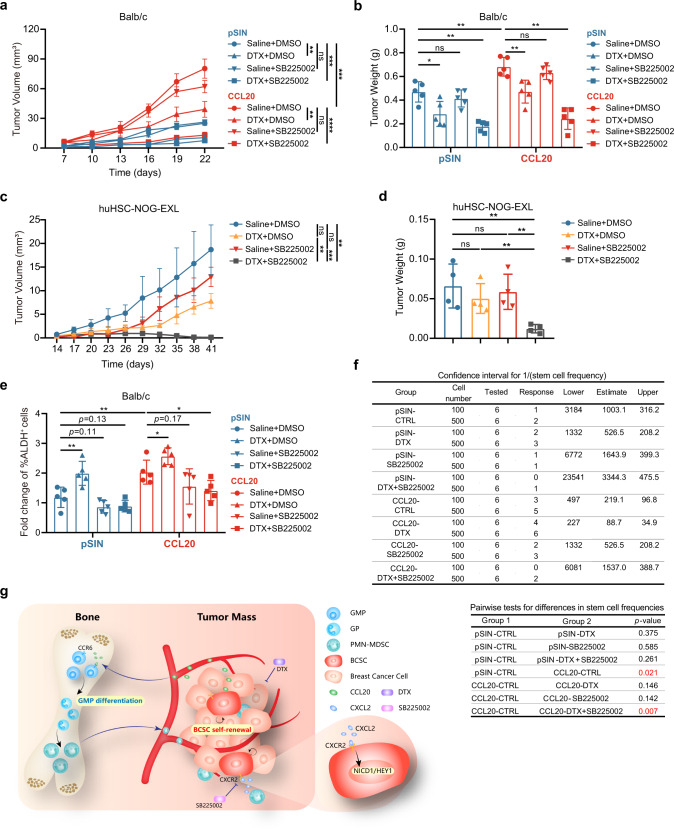
CXCR2 antagonist enhanced the therapeutic efficacy of docetaxel on breast tumors. **a, b** Balb/c mice were orthotopically transplanted with pSIN-/CCL20-overexpressing 4T1 cells (2×10^4^) at the fourth mammary fat pads for combinational treatment (*n* = 5 for each group). Tumor size was monitored every 3 days, and tumor volume was calculated (**a**). The tumor was weighed after mice were sacrificed (**b**). **c, d** huHSC-NOG-EXL mice were orthotopically transplanted with MDA-MB-231 cells (2 × 10^6^) at the fourth mammary fat pads (*n* = 4 for each group). Tumor size was monitored every 3 days, and tumor volume was calculated (**c**). The tumor was weighed after mice were sacrificed (**d**). **e** The percentage of ALDH^+^ BCSCs was determined by ALDEFLUOR assay in tumor cells from pSIN-/CCL20-overexpressing 4T1 cell allograft tumors and shown in bar graph as mean ± SEM. **f** Tumor cells (CD45^−^CD140b^−^CD31^−^) isolated from SB225002- and/or DTX-treated pSIN or CCL20-overexpressing tumors were engrafted to mammary fat pads of Balb/c mice at a limited dilution (*n* = 3 for each group, two sites each mouse, 100 or 500 cells/site). The stem cell frequency and *p*-value calculation were based on the positive tumor sites. **g** The schematic diagram for the findings of the current studies. CCL20 promoted GMP differentiation to GPs in BM by binding to CCR6, resulting in the PMN-MDSC expansion. CCL20-modulated PMN-MDSCs secreted amounts of CXCL2 and then activated NOTCH1/HEY1 signaling pathway via binding to CXCR2 to enhance the stemness of breast cancer cells. CXCR2 antagonist SB225002 combined with DTX in vivo not only dramatically inhibited the tumor growth but also significantly decreased the stemness of breast cancer cells. ns, no significance; *p < 0.05, **p < 0.01, ***p < 0.001, ****p < 0.0001

Given the critical role and clinical relevance of CCL20-modulated PMN-MDSCs on promoting stemness of breast cancer cells, we sought to evaluate whether targeting CXCL2-CXCR2 axis could be an effective strategy to treat CCL20^high^-expressing breast cancer patients who are prone to show poor response to DTX. In addition to the syngeneic mouse model, humanized hematopoietic stem cell-NOG-EXL (huHSC-NOG-EXL) mice, which express human interleukin-3 (hIL-3) as well as granulocyte-macrophage colony-stimulating factor (GM-CSF) and show in general a superior humanization phenotype,^44,45^ was constructed to elucidate the potential synergies of DTX and CXCR2 inhibition. We orthotopically transplanted pSIN-/CCL20-overexpressing 4T1 or Py8119 cells into the mammary fat pads of Balb/c or C57BL/6N mice, as well as MDA-MB-231 cells, which showed higher CCL20 expression (Supplementary Fig. S22), into the mammary fat pads of huHSC-NOG-EXL mice. We designed a novel treatment strategy by combining DTX with a potent and selective CXCR2 antagonist SB225002 in these orthotopic breast tumor mouse models^46,47^ (Supplementary Fig. S23a, b) and found that the combination of DTX and SB225002 showed a more significant inhibiting effect on tumor growth than either treatment alone in both syngeneic mice and humanized mice bearing CCL20^high^-expressing breast cancer cell allograft or xenograft tumors (Fig. 6a–d and Supplementary Figs. S23c, S24a–c). SB225002 treatment effectively decreased ALDH^+^ BCSCs (Fig. 6e and Supplementary Figs. S24d, S25). Consistently, the combinational treatment showed the inhibition on synergistic effects on tumor shrinkage (Supplementary Fig. S26) and secondary tumor initiation (Fig. 6f and Supplementary Fig. S24e).

These results showed that the CXCR2 antagonist obviously enhanced the therapeutic efficacy of DTX and might be a promising candidate to increase anticancer efficacy of DTX in breast cancer, especially in CCL20^high^-expressing breast cancer, suggesting the combinational treatment may be more effective in CCL20^high^-expressing breast cancer patients.

## Discussion

High CCL20 expression levels were positively correlated to tumor stage and grade, as well as the occurrence of pleural metastases in human triple-negative breast cancer cell lines.^25,48^ Moreover, previous study showed that CCL20 could promote BCSC self-renewal through the activation of nuclear factor kappa B (NF-κB) and p38 mitogen-activated protein kinase (MAPK) activity.^28^ Nevertheless, there is no evidence of a causative or functional link between TME regulated by CCL20 and breast cancer progression, particularly in BCSCs. In this study, we discovered for the first time that CCL20 modulated the PMN-MDSC expansion in BM, blood, spleen and tumor, promoting tumor immune-suppressive microenvironment. Mechanistically, CCL20 promoted the GMP differentiation to GPs via binding to its receptor CCR6, contributing to the PMN-MDSC expansion. GMPs have been found to be increased across a variety of tumor types in previous studies.^20^ Poorer clinical outcomes were associated with higher circulating GMPs, as reflected by clinical stages and reduced time to progression.^20^ Consistent with this process is our observation that GMPs in CCL20-overexpressing cell allograft mouse model were remarkably increased. It has been reported that lineage-instructive transcription factors, such as IFN regulatory factor 8 (IRF8), regulated the production and differentiation of GMPs.^35,49^ However, the key molecules that influence GMP differentiation are poorly studied in cancer progression. In this study, we identified that the differential direction of GMPs was influenced by CCL20. CCL20 stimulated the GMP differentiation to GPs as resulting in the PMN-MDSC expansion in BM. These studies confirmed CCL20 as an important positive regulator of PMN-MDSC production. In future, concrete mechanisms on how CCL20-CCR6 axis influenced the differentiation of GMPs to GPs need to be elucidated.

In the interactions between tumor cells and immune cells, the TME is the main battleground. In recent years, a great deal of research has focused on how the immune system modulates CSCs.^50^ Deregulated differentiation of myeloid-derived cells was reported to alter tumor phenotype by changing the plasticity of BCSCs.^51,52^ There is considerable evidence that PMN-MDSCs promote cancer cell stemness via numerous ways, including S100A9-positive exosome, piRNA-823 expression, DNMT3B activation, and IL-6/STAT3 and NO/NOTCH cross-talk signaling.^13,23,24^ In this study, we have yielded significant original insights into specific immunobiology and pathology between CCL20-modulated PMN-MDSCs and CSCs in the context of breast cancer. The tumorsphere formation ability was increased, and aldehyde dehydrogenase and the levels of a panel of established CSC markers, krüppel-like factor 4 (KLF4), nanog homeobox (NANOG), SRY-box transcription factor 9 (SOX9), and ALDH1A1 were all elevated in breast cancer cells co-cultured with CCL20-moducated PMN-MDSCs. Furthermore, due to the accumulation of PMN-MDSCs and enrichment of BCSCs, immunosuppressed microenvironments are formed to contribute to suppress the CD8^+^IFNγ^+^ T cell activity. These changes caused by CCL20-overexpression lead to a feedback loop that is highly effective in promoting tumor progression, although further studies are required to clarify whether there is a specific subtype of PMN-MDSCs modulated by CCL20 to drive this loop.

We investigated the key mediator in CCL20-modulated PMN-MDSCs that conveyed breast cancer cell stemness. Bioinformatic analysis showed that CXCL2 was positively related to CCL20 in breast cancer patients (*p* value = 8.4e-31, R = 0.34) (Supplementary Fig. S27), and CXCL2 was abundant and highly secreted by PMN-MDSCs in CCL20-overexpressing tumors. Considerable evidence showed that CXCL2 overexpression was poor prognosis in patients with ovarian cancer, cervical cancer, etc.^53,54^ The upregulated CXCL2 expression in cancer cells was proposed to promote invasion and migration.^55^ Here, we demonstrated CXCL2 secreted by PMN-MDSCs in TME cooperated with its receptor CXCR2 which was highly expressed on BCSCs, indicating a cross-talk between PMN-MDSCs and BCSCs was achieved by CXCL2-CXCR2 axis. In addition, we observed the phenomenon that CXCL2 increased CXCR2 expression in vitro and in vivo. In addition, CXCR2 expression was higher in ALDH^+^ BCSCs compared to ALDH^−^ BCSCs (Supplementary Fig. S15). We speculated that the enrichment of ALDH^+^ BCSCs induced by CXCL2 might contribute to the increased CXCR2 expression, which provided a positive feedback loop to promote the stemness of breast cancer cells. Specific mechanisms deserve to be further explored.

DTX is one of the most widely used antimitotic chemotherapy drugs for the treatment of cancers.^56^ It has been reported that DTX possesses chemo-immunomodulatory properties besides its canonical antitumor properties.^57,58^ Our recent reports showed that DTX induced C-C motif chemokine ligand 3 (CCL3) secretion in macrophages and triggered proinflammatory polarization of macrophages to inhibit breast cancer progression.^59^ However, most patients eventually become chemo-resistant accompanied with tumor progression. There is considerable evidence that CSC enrichment played a key role in contributing to chemotherapy resistance of DTX.^28,60^ CXCR2 was reported to induced epithelial-to-mesenchymal transition (EMT) of breast cancer cells and proposed as a novel marker of BCSCs.^61–63^ In our study, we used SB225002, a selective CXCR2 antagonist with promising therapeutic effect in cancers,^46^ to blocking CXCR2 in breast cancer cells. Our results demonstrated that CXCR2 blockade sensitized cancer cells to DTX via decreasing cancer cell stemness. Additionally, due to the increasing accumulation of PMN-MDSCs and enrichment of ALDH^+^ BCSCs, the combination of DTX and CXCR2 antagonist had a better therapeutic effect in CCL20-overexpressing cell allograft mouse model. Collectively, these results indicated that CXCR2 blockade augmented chemotherapeutic effects of DTX, especially in CCL20^high^-expressing breast cancer patients.

In conclusion, we discovered that the CCL20 overexpression in breast cancer cells significantly promoted the PMN-MDSC expansion. The CCL20-modulated PMN-MDSCs in TME played indispensable roles in promoting breast cancer cell stemness. CXCR2 antagonist SB225002 combined with DTX may be a hopeful therapeutic strategy to overcome chemoresistance and gain better clinical outcomes in patients with breast cancer, especially CCL20^high^-expressing breast cancer (Fig. 6g).

## Materials and methods

### Ethics statements

The Shanghai Cancer Center in Fudan University provided the tumor tissues for all the breast cancer patients. For each patient involved, informed consent was obtained. The study was approved by Fudan University Shanghai Cancer Center Institutional Review Board (050432-4-1212B).

The animal experiments were strictly carried out in accordance with the People’s Republic of China Legislation Regarding the Use and Care of Laboratory Animals and approved by the Fudan University Shanghai Cancer Center Institutional Review Board (JS-082).

### Cell culture

The breast cancer cell lines MDA-MB-231, 4T1, and Py8119 were purchased from ATCC. The culture medium for MDA-MB-231 and 4T1 was RPMI 1640 medium (Gibco) supplemented with 5% fetal bovine serum (FBS) (Gibco) and 1% penicillin/streptomycin (Beyotime Biotechnology). The culture medium for Py8119 cells was F12 medium (Gibco) with 5% FBS (Gibco), 1 µg/ml hydrocortisone (Sigma), 50 µg/ml gentamicin (Sigma), 10 ng/ml epidermal growth factor (PeproTech) and 5 µg/ml insulin (Biosharp Life Science). HEK293T cells were cultured in DMEM (Gibco) medium supplemented with 10% FBS and 1% penicillin/streptomycin. All cell lines were evaluated and authenticated. These cell lines were maintained at 37 °C in a humidified atmosphere of 5% CO_2_ incubator.

### Plasmid/short hairpin RNA (shRNA) construction and lentivirus transfection

*CCL20* was amplified using the complementary DNA (cDNA) of 4T1 cell line and cloned into pSIN-puro lentiviral vector (Addgene), and the authenticity was verified by sequencing. The shRNA sequence of CXCR2 from Sigma-Aldrich was cloned into pLKO.1-puro lentiviral vector (Addgene). Then, HEK293T cells were transfected with plasmid DNA to generate high titer lentivirus which was used to infect breast cancer cells to establish stable cell lines. The primers used for plasmid construction were listed in Supplementary Table 1.

### Mice and tumor models

Female Balb/c mice, C57BL/6N mice as well as the huHSC-NOG-EXL were purchased from Charles River and housed in specific pathogen-free facilities at the Department of Laboratory Animal Science of Fudan University. For the determination of tumorigenicity, 4T1 cells (5 × 10^4^), Py8119 cells (1 × 10^5^) or MDA-MB-231 cells (2 × 10^6^) were orthotopically injected into the fourth mammary fat pads of Balb/c mice, C57BL/6N mice or huHSC-NOG-EXL mice, respectively. Tumor size was measured every 3 days and calculated as tumor volume = Length × Width^2^/2.

CXCR2 antagonist SB225002 (Selleckchem) was suspended in special solvent that consisted of 30% polyethylene glycol, 5% Tween-80, 2% dimethyl sulfoxide, and 63% water. For the combination therapy of SB225002 and docetaxel (DTX) (Hengrui) in vivo, mice were randomly separated into the indicated groups when the average diameter of tumors reached approximately 2–3 mm. SB225002 (10 mg/kg, once every 3 days) or equal volume vehicle alone or combined with DTX (20 mg/kg for 4T1, 40 mg/kg for Py8119, once every 6 days) were administered by intraperitoneal injection.

### RNA isolation and quantitative real-time PCR

Total RNA from samples was extracted with TRIzol reagent (Takara), and the cDNA was obtained by reverse transcription using the HiScript II 1st Strand cDNA Synthesis kit (Vazyme Biotech) and T100 Thermal Cycler (BIO-RAD). The quantitative real-time PCR (qRT-PCR) was performed with AceQ Universal SYBR qPCR Master Mix (Vazyme Biotech) using 7300Plus Real-Time PCR System (Applied Biosystems) according to the manufacturer’s instructions. The mRNA level of each gene was expressed relative to reference gene. Relative expression value was calculated by using the comparative C_t_ method (2^−ΔCt^). The primers used for qRT-PCR were listed in Supplementary Table 2.

### ELISA

The conditioned medium of established breast cancer cell lines and the blood serum of mice bearing breast cancer cell orthotopic allograft tumors were collected to determine CCL20 levels according to the manufacturer’s instructions (RayBiotech). For the detection of CXCL2, PMN-MDSCs sorted from tumors were cultured for 2 days in vitro. The CXCL2 levels in the conditioned medium of PMN-MDSCs were determined with a Mouse CXCL2 ELISA Assay kit (RayBiotech) according to the manufacturer’s instructions.

### Western blotting

Cells were lysed with RIPA buffer (Beyotime Biotechnology) containing phenylmethylsulfonyl fluoride (PMSF) (Beyotime Biotechnology) on ice for 30 min. The protein lysates were quantified with a BCA kit (Thermo Fisher) and then denatured in loading buffer. Equal amounts of lysates were electrophoretically separated by sodium dodecyl sulfate-polyacrylamide gel electrophoresis (SDS-PAGE) and subsequently transferred onto polyvinylidene fluoride (PVDF) membranes (Millipore). 5% de-fat milk was used for blocking the membranes at room temperature (RT) for 1 h. Then, they were incubated with primary antibody at 4 °C overnight, followed by the incubation with HRP-conjugated secondary antibody at RT for 1 h. Chemiluminescence was detected to use an ImageQuant LAS 4000 Micro Imaging System (GE) with western HRP Substrate (Millipore). Intensities in the resulting bands were quantified by ImageJ software. The antibody information used for western blotting was shown in Supplementary Table 3.

### Isolation of breast cancer cells and immune cells, flow cytometry and cell sorting

Single-cell suspensions were made from tumor tissues and different organs of mice bearing breast cancer cell orthotopic allograft tumors. For analysis of tumor and spleen, single-cell suspensions were prepared as previously described.^28^ For BM analysis, hind limb bones of mice (femurs and tibias) were cut off. After removing the fur and muscles, bones were flushed using a 1 ml sterile syringe, and then the obtained cells were passed through a 70 μm filter. For blood analysis, blood samples were collected in EDTA-coated tubes and centrifuged at 1200 rpm for 10 min to separate serum and plasma. The erythrocytes in plasma were lysed by ACK lysis buffer (BioLegend).

When analyzing cancer cells, PE-conjugated anti-mouse CD45, CD31 and CD140b antibodies were used. For immune cell analysis, single-cell suspensions were firstly incubated with anti-mouse CD16/32 antibody to block the non-specific binding. To analyze the percentage of total MDSCs with its two subtypes PMN-MDSCs and M-MDSCs, the cells were labeled with fluorescence-conjugated antibodies to CD45, CD11b, Gr-1, Ly6C and Ly6G. To identify the subtypes of T cells, the cells were stained with CD45, CD3, CD8, followed by the intracellular staining of IFNγ. Macrophages were analyzed using antibodies to CD45, CD11b, F4/80, MHCII and CD206. For the identification of bone marrow progenitor cells, bone marrow cells were labeled with lineage marker (CD3, CD4, CD8a, CD19, CD45R/B220, CD127) and fluorescence-conjugated antibodies to c-Kit, Sca-1, CD34, FcγR, Ly6C and CD115. The viability of cells was analyzed by DAPI (Sigma) staining. The antibody information used for flow cytometries was shown in Supplementary Table 3.

For ALDEFLUOR assay (StemCell Technologies), dissociated single cells were resuspended in ALDEFLUOR buffer containing ALDEFLUOR substrate BAAA and incubated at 37 °C for 40 min. DEAB was added as a negative control. All the flow cytometry and cell sorting experiments were performed using MoFlo Astrios or CytoFlex instrument (Beckman Coulter) and analyzed by Summit 6.3 software.

### Immunohistochemistry (IHC) and Immunofluorescence (IF) staining

For IHC staining, the tumor tissues from patients or mice were fixed in formalin, dehydrated with graded alcohols, and then embedded in paraffin. Sectioned samples were deparaffined in xylene and rehydrated. Endogenous peroxidase was inactivated using 3% H_2_O_2_ diluted in methanol, and antigen retrieval was performed with citric acid under high temperature and high pressure. The sections were then blocked with animal non-immune serum (Maixin Biotech) and incubated with primary antibodies at 4 °C overnight. After washing, the sections were incubated with secondary antibodies at RT for 30 min and stained with DAB detection kit (MaxVision). Hematoxylin (ZSGB-BIO) was used for cell nucleus staining, and the visualization was achieved by microscope (OLYMPUS BX43). For IF staining, the sectioned samples were basically subjected to the same experimental operations as above. DAPI (Invitrogen) was used for cell nucleus staining, and the images were observed and captured by confocal microscope (Zeiss LSM710). The antibody information was shown in Supplementary Table 3.

### Colony-forming unit assay

To evaluate the myeloid differentiation potential of bone marrow progenitor cells, GMPs and GPs (1000 cells/well) sorted from BM of Balb/c mice bearing 4T1 pSIN-/CCL20-overexpressing cell orthotopic allograft tumors, or sorted from BM of Balb/c mice bearing parental 4T1 cell orthotopic allograft tumors with rmCCL20 (10 ng/ml, R&D systems) in the culture using MethoCult GF M3434 (STEMCELL Technologies) in 96-well ultra-low attachment plates (Corning) for 10 days. Fresh methylcellulose-based medium was added every 2–3 days. The colonies were identified and counted by microscope (Olympus IX73).

### RNA interference

Murine CCR6-siRNA and non-silencing scrambled control (SCR) siRNA were purchased from GenePharma Biotech. The sequences corresponding to the indicated siRNA were as follows: CCR6-siRNA, 5’-GUGUAUGAGAAGGAAGAAUAAdTdT-3’, 3’-UUAUUCUUCCUUCUCAUACACdTdT-5’; SCR-siRNA, 5’-UUCUCCGAACGUGUCACGUdTdT-3’, 3’-ACGUGACACGUUCGGAGAAdTdT-5’. Cells were cultured in serum-free medium and transfected with the a mixture of siRNA and Lipofectamine^TM^ 3000 Transfection Reagent (Invitrogen) according to the manufacturer’s instructions. After incubation at 37 °C for 4 h, fresh culture medium supplemented with 10% FBS was added to the cells and the appropriate treatment was performed.

### Mammosphere formation assay

Murine breast cancer cells (200 cells/well) were mix-cultured with PMN-MDSCs sorted from tumors in 96-well ultra-low attachment plates (Corning) for 10 days. The MammoCult Human Medium kit (StemCell Technologies) supplemented with 4 μg/ml heparin (StemCell Technologies), 1 μg/ml hydrocortisone (Sigma), and 1% pen-strep antibiotic (Beyotime Biotechnology) was required. Fresh complete mammocult medium was added every 3–4 days. After culture was completed, spheres were collected by centrifugation at 500 rpm for 5 min and digested with 0.25% trypsin at 37 °C for 5 min. Single cells were centrifuged and resuspended for subsequent experiments. Flow cytometry was used to distinguish breast cancer cells and PMN-MDSCs with specific cell surface markers. The images of mammospheres were observed by microscope (Olympus IX73).

### Co-culture or mix-culture of PMN-MDSCs and breast cancer cells

PMN-MDSCs (1 × 10^6^ cells/well) were sorted from tumors and mix-cultured or co-cultured with the corresponding breast cancer cells (2 × 10^5^ cells/well) in 6-well plates for 3 days. Transwell insert with pore size of 0.4 µm (Corning) was applied for cell co-culture. PMN-MDSCs and breast cancer cells were placed in the lower and upper chambers of the insert respectively. Meanwhile, PMN-MDSCs from tumors were co-cultured or mix-cultured with scramble or CXCR2-knockdown 4T1 cells in the same method.

### In vitro limiting dilution assay

The murine breast cancer cell lines 4T1 or Py8119 were treated with rmCXCL2 (10 ng/ml) or co-cultured with pSIN^PMN^ or CCL20^PMN^ from pSIN-/CCL20-overexpressing 4T1 or Py8119 cell allograft tumors for 3 days. Then, 4T1 cells were seeded at a density of 5, 10, 20, 50, 100 cells per well and Py8119 cells were seeded at a density of 25, 50, 100, 250, 500 cells per well in 96-well ultra-low attachment plates with complete mammocult medium. Fresh culture medium was added every 3–4 days, and the mammosphere formation was observed after 10 days. The BCSC frequency was calculated by the Extreme Limiting Dilution Analysis (ELDA, http://bioinf.wehi.edu.au/software/elda).

### Soft agar colony formation assay

Breast cancer cells (8000 cells/well) were resuspended in culture medium containing 0.3% low melting agarose (Sangon Biotech) and overlaid with 0.6% low melting agarose in 6-well plates. After incubation for 4–6 weeks, colonies were stained with 0.005% crystal violet, and then the number of colonies was analyzed.

### RNA sequencing

For RNA sequencing (RNA-Seq), breast cancer cells and PMN-MDSCs were obtained from tumors by flow cytometry, and 4T1 cells were treated with rmCCL20 (10 ng/ml, R&D systems) or PBS for 3 days in vitro. Total RNA of sorted cells and cell lines were extracted with TRIzol reagent (Takara Bio). RNA-Seq libraries were established using the NEB Next Ultra Directional RNA Library Prep kit for Illumina (New England Biolabs) and checked by quality control with 2100 Bioanalyzer (Agilent). Sequencing was performed on HiSeq3000 platform (Illumina). The RNA-Seq data were subjected to unsupervised clustering and transformed into heat maps. Enrichment pathway analysis of genes was compiled from both GSEA and Metascape databases, and a *p* value < 0.05 was considered statistically significant.

### Statistical analysis

All data were presented as the mean ± SEM and analyzed by GraphPad Prism 8.0. Unless otherwise indicated, comparisons between two data groups were performed with unpaired, two-tailed Student’s *t* test. Two-way ANOVA was used for multiple comparisons. *P* values were considered statistically significant as follows: *p < 0.05, **p < 0.01, ***p < 0.001 and ****p < 0.0001.

## Supplementary information

Revised Supplementary Materials

## Data Availability

RNA-seq data profiles from this study have been deposited in the NCBI under accession code PRJNA911328. All other data supporting the results can be found in this paper and its supplementary materials. All other relevant data can be obtained from the corresponding authors upon request.
