# Supplementary material for: PMN-MDSCs modulated by CCL20 from cancer cells promoted breast cancer cell stemness through CXCL2-CXCR2 pathway
Source: Signal Transduct Target Ther. 2023 Mar 1;8:97. doi: 10.1038/s41392-023-01337-3 (PMC9977784; doi:10.1038/s41392-023-01337-3)
Supplement: Supplementary file 1 — Revised Supplementary Materials [file 41392_2023_1337_MOESM1_ESM.docx]

Supplementary Materials for

PMN-MDSCs modulated by CCL20 from cancer cells promoted breast cancer cell stemness through CXCL2-CXCR2 pathway

Rui Zhang^1#^, Mengxue Dong^1#^, Juchuanli Tu^1^, Fengkai Li^1^, Qiaodan Deng^1^, Jiahui Xu^1^, Xueyan He^1^, Jiajun Ding^1,2^, Jie Xia^1^, Dandan Sheng^1^, Zhaoxia Chang^1^, Wei Ma^1^, Haonan Dong^1^, Yi Zhang^3^, Lixing Zhang^1^, Lu Zhang^1*^, Suling Liu^1,4*^

^1^Fudan University Shanghai Cancer Center & Institutes of Biomedical Sciences; State Key Laboratory of Genetic Engineering; Cancer Institutes; Key Laboratory of Breast Cancer in Shanghai; The Shanghai Key Laboratory of Medical Epigenetics; Shanghai Key Laboratory of Radiation Oncology; The International Co-laboratory of Medical Epigenetics and Metabolism, Ministry of Science and Technology; Shanghai Medical College; Fudan University, Shanghai 200032, China

^2^Breast Surgery, Obstetrics and Gynecology Hospital of Fudan University, Shanghai, China

^3^Department of Breast and Thyroid Surgery, Southwest Hospital, the First Affiliated Hospital of the Army Military Medical University, Chongqing 400038, China

^4^Jiangsu Key Lab of Cancer Biomarkers, Prevention and Treatment, Collaborative Innovation Center for Cancer Medicine, Nanjing Medical University, Nanjing 211166, China

*Corresponding authors

^#^Rui Zhang and Mengxue Dong contributed equally.

Correspondence to: [suling@fudan.edu.cn;](mailto:suling@fudan.edu.cn;) [zlu7025@gmail.com](mailto:zlu7025@gmail.com)

**This PDF file includes:**

Figures S1 to S27

Tables S1 to S3

**Other Supplementary Materials for this manuscript include:**

Data S1. The raw data of Western blots

Data S2. Gating strategies for the flow cytometries

**
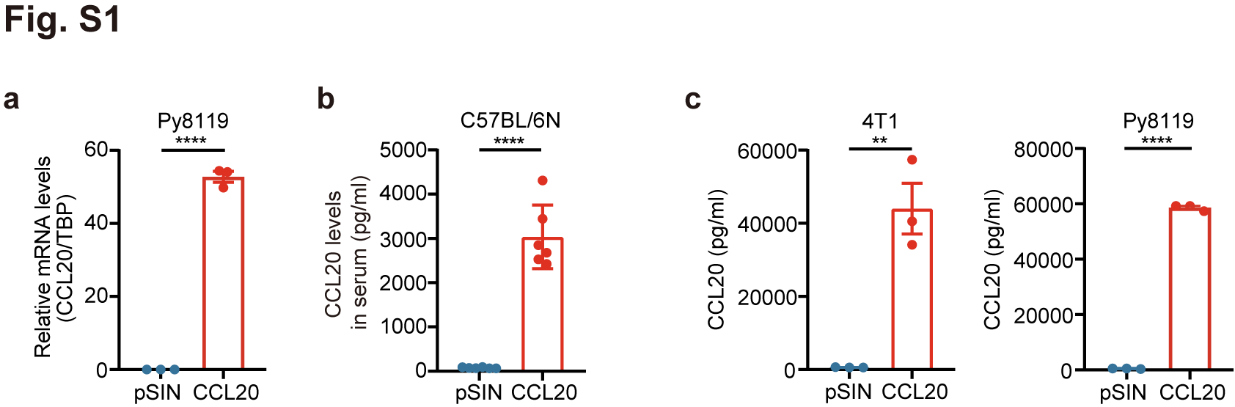
**

Fig. S1. The establishment of CCL20-overexpressing cell lines and corresponding mouse models.

**a** pSIN-/CCL20-overexpressing Py8119 cell lines were established, and overexpression efficiency of CCL20 was confirmed by qRT-PCR. Bar graph was presented as the mean of three biological independent experiments (mean ± SEM). C57BL/6N mice were orthotopically transplanted with pSIN-/CCL20-overexpressing Py8119 cells (1×10^5^) at the fourth mammary fat pads. **b** The level of CCL20 in the blood serum of C57BL/6N mice was measured by ELISA, and bar graph was shown as mean ± SEM. **c** The amount of CCL20 in culture medium of pSIN-/CCL20-overexpressing 4T1 (left) and Py8119 (right) was measured by ELISA. Bar graph was presented as the mean of three biological independent experiments. ***p* < 0.01, *****p* < 0.0001.


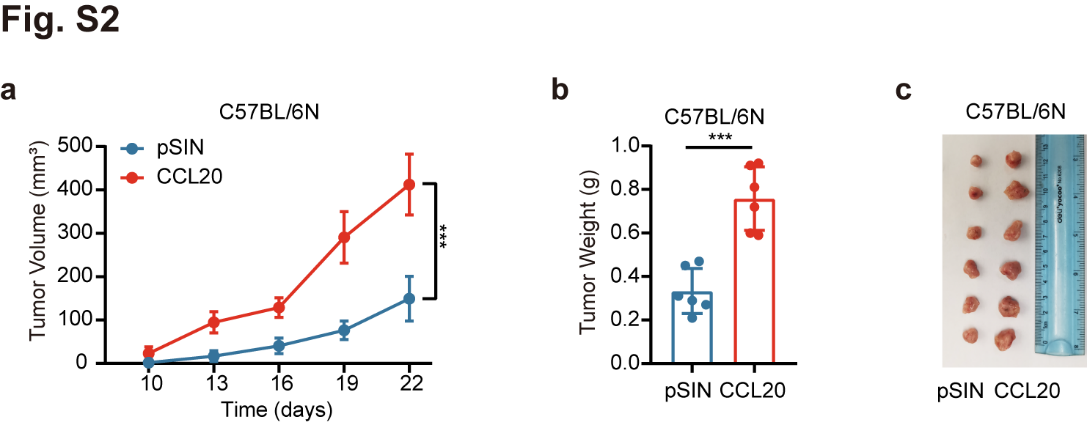


Fig. S2. CCL20 promoted tumor growth in C57BL/6N mice bearing Py8119 cell allograft tumors.

C57BL/6N mice were orthotopically transplanted with pSIN-/CCL20-overexpressing Py8119 cells (1×10^5^) at the fourth mammary fat pads (*n*=6 for each group). **a** Tumor size was monitored every 3 days and tumor volume was calculated. **b, c** Tumor weight (b) and tumor image (c) was shown after the mice were sacrificed**.** ****p* < 0.001.


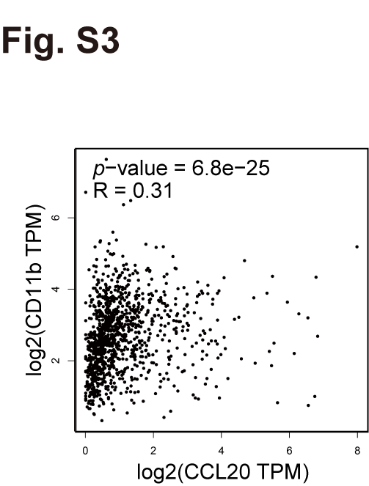


Fig. S3. The correlation between CCL20 expression and CD11b expression in patients’ breast cancer.

CCL20 mRNA expression was positively correlated with the mRNA expression of CD11b (a myeloid marker in human) in GEPIA2 database (http://gepia2.cancer-pku.cn/#index).


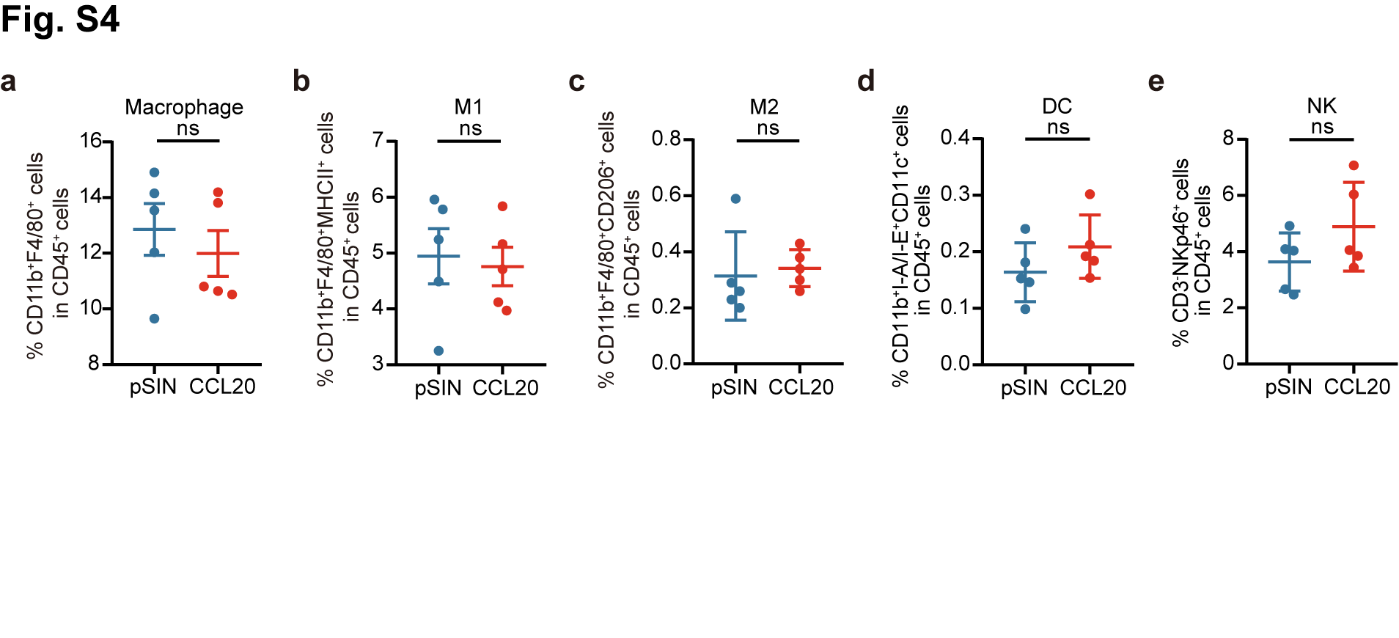


Fig. S4. CCL20 had no effects on the percentage of macrophages, DCs, and NK cells in tumors.

Balb/c mice were orthotopically transplanted with pSIN-/CCL20-overexpressing 4T1 cells (5×10^4^) at the fourth mammary fat pads (*n*=5 for each group). **a-e** The percentage of total macrophage (CD45^+^CD11b^+^F4/80^+^) (a), M1-like macrophage (CD45^+^CD11b^+^F4/80^+^MHCII^+^) (b), M2-like macrophage (CD45^+^CD11b^+^F4/80^+^CD206^+^) (c), DCs (CD45^+^CD11b^+^I-A/I-E^+^CD11c^+^) (d), and NK cells (CD45^+^CD3^-^NKp46^+^) (e) in pSIN-/CCL20-overexpressing 4T1 cell allograft tumors were analyzed by flow cytometry and shown in bar graph as mean ± SEM. ns, no significance.


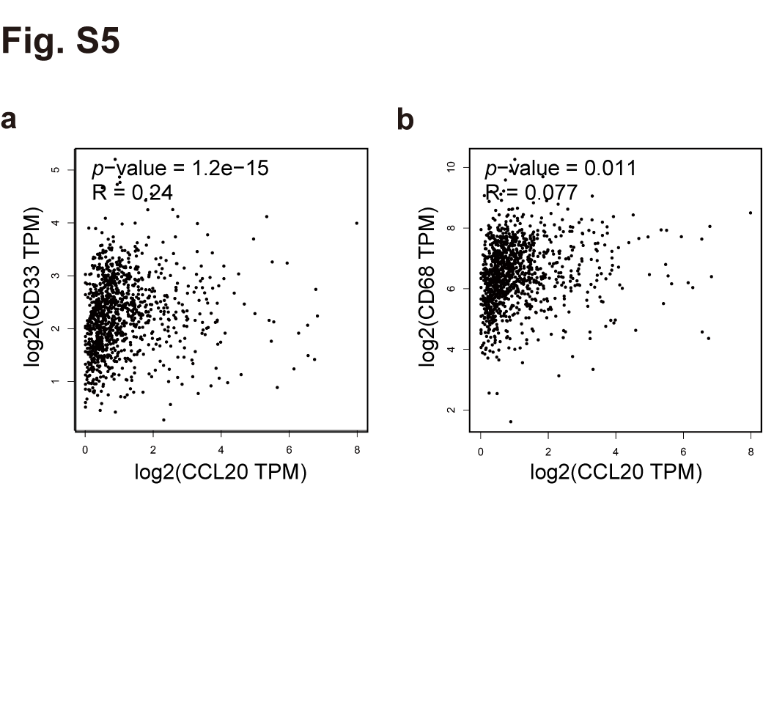


Fig. S5. The correlation between CCL20 expression and the expressions of myeloid immune cell markers in patients’ breast cancer.

**a, b** CCL20 mRNA expression was positively correlated with the mRNA expression of CD33 (a MDSC marker in human) (a), but not CD68 (a pan-macrophage marker in human) (b) in GEPIA2 database (http://gepia2.cancer-pku.cn/#index).


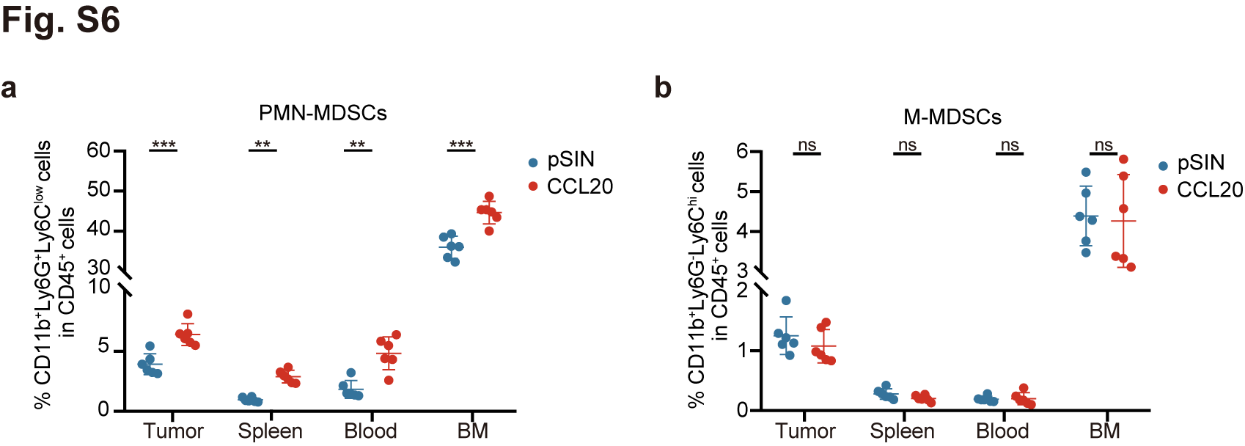


Fig. S6. The PMN-MDSC percentage was significantly increased in C57BL/6N mice bearing CCL20-overexpressing Py8119 cell allograft tumors.

**a, b** The percentage of PMN-MDSCs (a) and M-MDSCs (b) in tumor, spleen, blood, and BM of C57BL/6N mice bearing pSIN-/CCL20-overexpressing Py8119 cell allograft tumors were analyzed by flow cytometry and shown in bar graph as mean ± SEM. ns, no significance; ***p* < 0.01, ****p* < 0.001.

**
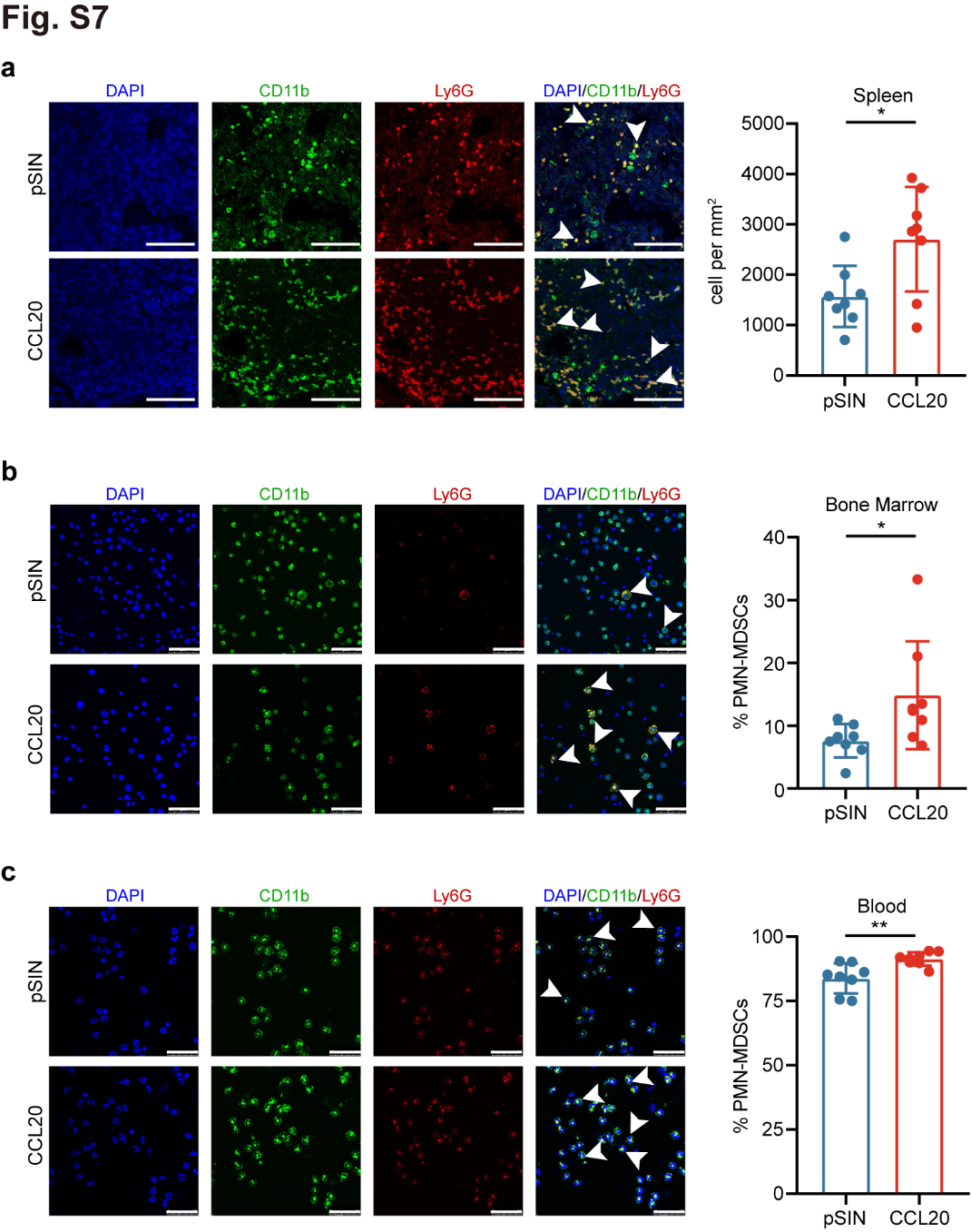
**

Fig. S7. The PMN-MDSC percentage was significantly increased in spleen, bone marrow, and blood from Balb/c mice bearing CCL20-overexpressing 4T1 cell allograft tumors.

**a-c** The percentage of CD11b-positive cells and Ly6G-positive cells in spleen (a), bone marrow (b), and blood (c) of Balb/c mice bearing pSIN-/CCL20-overexpressing 4T1 cell allograft tumors were analyzed by IF staining. Scale bar, 50 μm. **p* < 0.05, ***p* < 0.01.


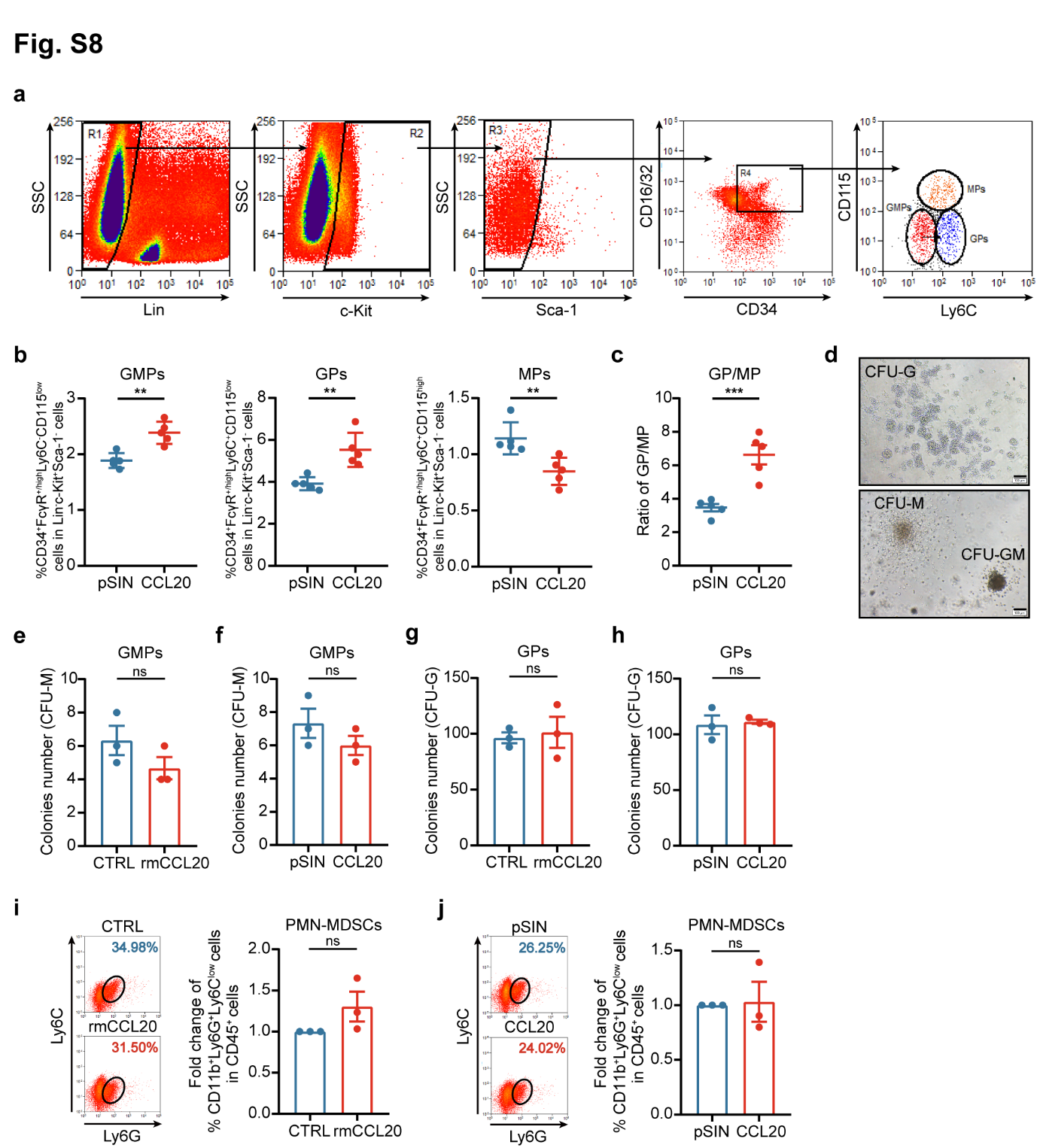


Fig. S8. CCL20 promoted PMN-MDSC expansion by inducing GMP differentiation to GPs.

**a** The gating strategy for sorting BM progenitor cells, including GMPs, GPs, and MPs by flow cytometry. Each type of progenitor cells was gated as follows: Lin^-^c-Kit^+^Sca-1^-^CD34^+^FcγR^+/high^Ly6C^-^CD115^low^ for GMPs, Lin^-^c-Kit^+^Sca-1^-^CD34^+^FcγR^+/high^Ly6C^+^CD115^low^ for GPs and Lin^-^c-Kit^+^Sca-1^-^CD34^+^FcγR^+/high^Ly6C^+^CD115^high^ for MPs. **b, c** C57BL/6N mice were orthotopically transplanted with pSIN-/CCL20-overexpressing Py8119 cells (1×10^5^) at the fourth mammary fat pads (*n*=5 for each group). After 21 days, mice were sacrificed, the percentage of BM progenitor cell populations including GMPs, GPs and MPs, were analyzed by flow cytometry (b) and the ratio of GP/MP was calculated (c). **d** Three types of BM progenitor cell colonies (CFU-G, CFU-M, and CFU-GM) were detected in CFU culture, and the representative images of colonies were shown. Scale bar, 100 μm. **e, f** GMPs were sorted from BM of mice bearing 4T1 cell allograft tumors and treated with rmCCL20 (10 ng/ml) (e), or were sorted from BM of mice bearing pSIN-/CCL20-overexpressing 4T1 cell allograft tumors (f). One thousand GMPs per well were cultured in the methylcellulose-based medium for 10 days. The number of CFU-M was counted and shown in bar graph as mean ± SEM. **g-j** GPs were sorted from BM of mice bearing 4T1 cell allograft tumors and treated with rmCCL20 (10 ng/ml) (g), or were sorted from BM of mice bearing pSIN-/CCL20-overexpressing 4T1 cell allograft tumors (h). One thousand GPs per well were cultured in the methylcellulose-based medium for 10 days. The number of CFU-G was counted and shown in bar graph as mean ± SEM. Cells from GP-derived colonies from g (i) or h (j) were collected and the PMN-MDSC percentage was analyzed by flow cytometry. Data were presented as mean ± SEM of three biological independent experiments. ns, no significance; ***p* < 0.01, ****p* < 0.001.


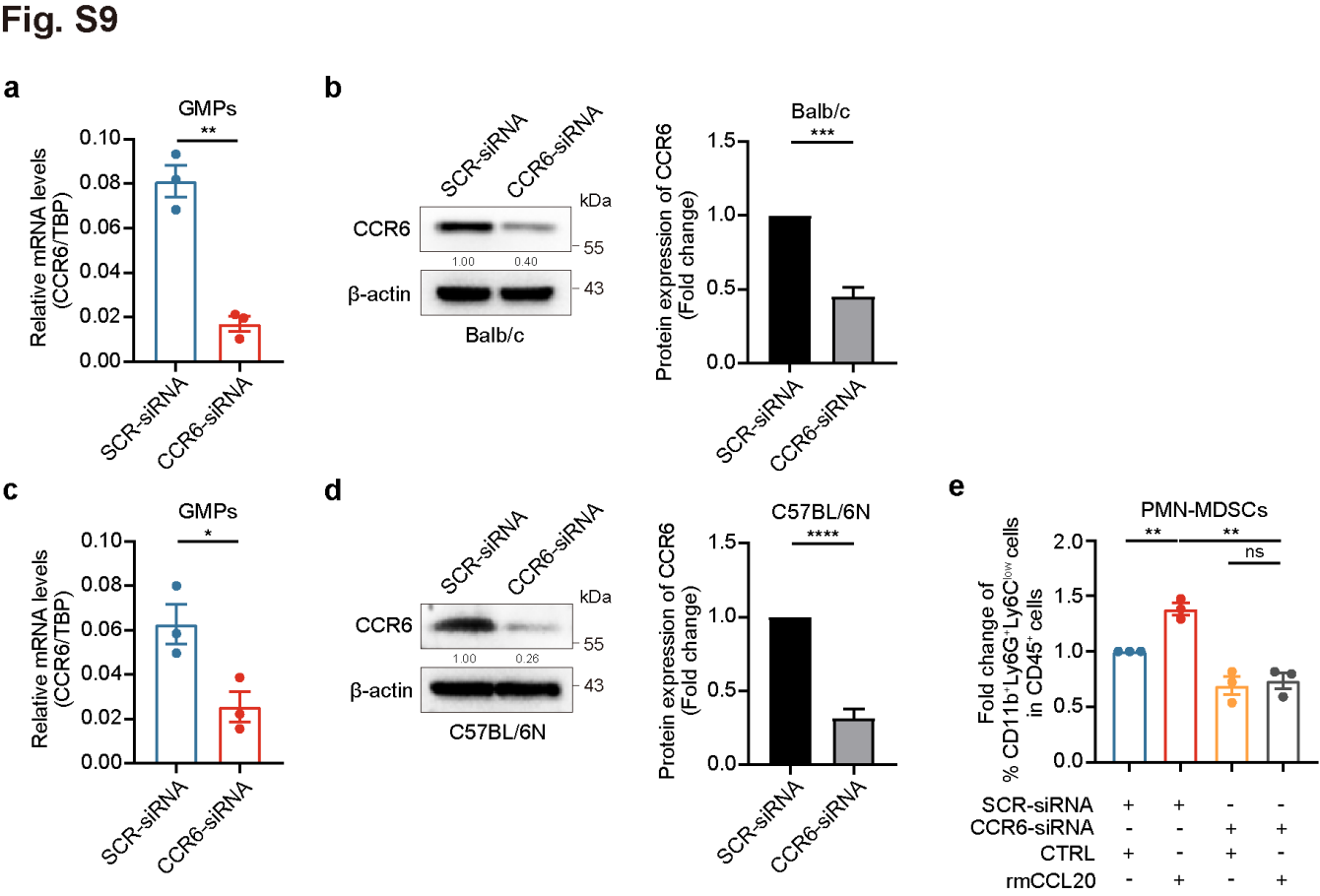


Fig. S9. CCL20 regulated GMP differentiation by binding to its receptor CCR6.

**a, b** GMPs were sorted from BM of mice bearing 4T1 cell allograft tumors and then transiently transfected with CCR6-siRNA. The knockdown efficiency of CCR6 in GMPs was analyzed by both qRT-PCR (a) and western blotting (b). β-actin was utilized as the internal control. **c-e** GMPs were sorted from BM of mice bearing Py8119 cell allograft tumors. After being transiently transfected with CCR6-siRNA, GMPs were treated with rmCCL20 (10 ng/ml) for 10 days. The knockdown efficiency of CCR6 in GMPs was analyzed by both qRT-PCR (c) and western blotting (d). β-actin was utilized as the internal control. The PMN-MDSC percentage was analyzed by flow cytometry (e). Bar graph was presented as the mean of three biologically independent experiments (mean ± SEM). ns, no significance; **p* < 0.05, ***p* < 0.01, ****p* < 0.001, *****p* < 0.0001.

**
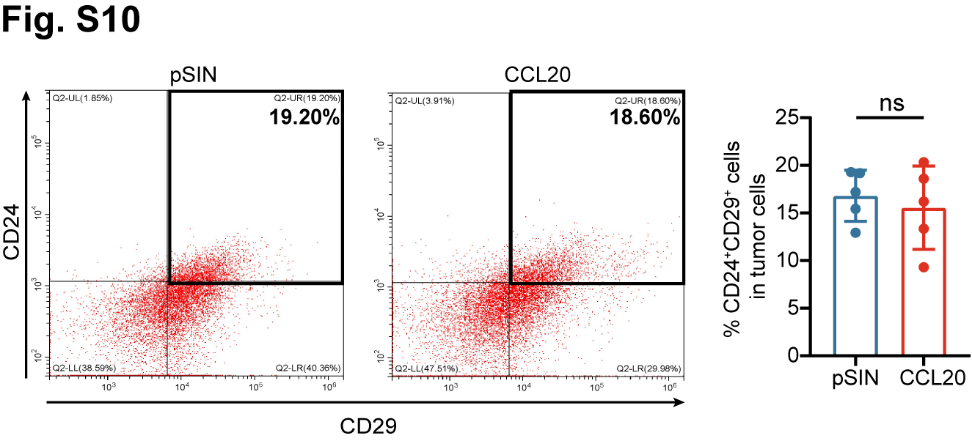
**

Fig. S10. CCL20 overexpression had no effects on the percentage of CD24^+^CD29^+^ BCSCs in tumors.

Balb/c mice were orthotopically transplanted with pSIN-/CCL20-overexpressing 4T1 cells (5×10^4^) at the fourth mammary fat pads (*n*=5 for each group). The percentage of CD24^+^CD29^+^ BCSCs in pSIN-/CCL20-overexpressing 4T1 cell allograft tumors was analyzed by flow cytometry and shown in bar graph as mean ± SEM. ns, no significance.


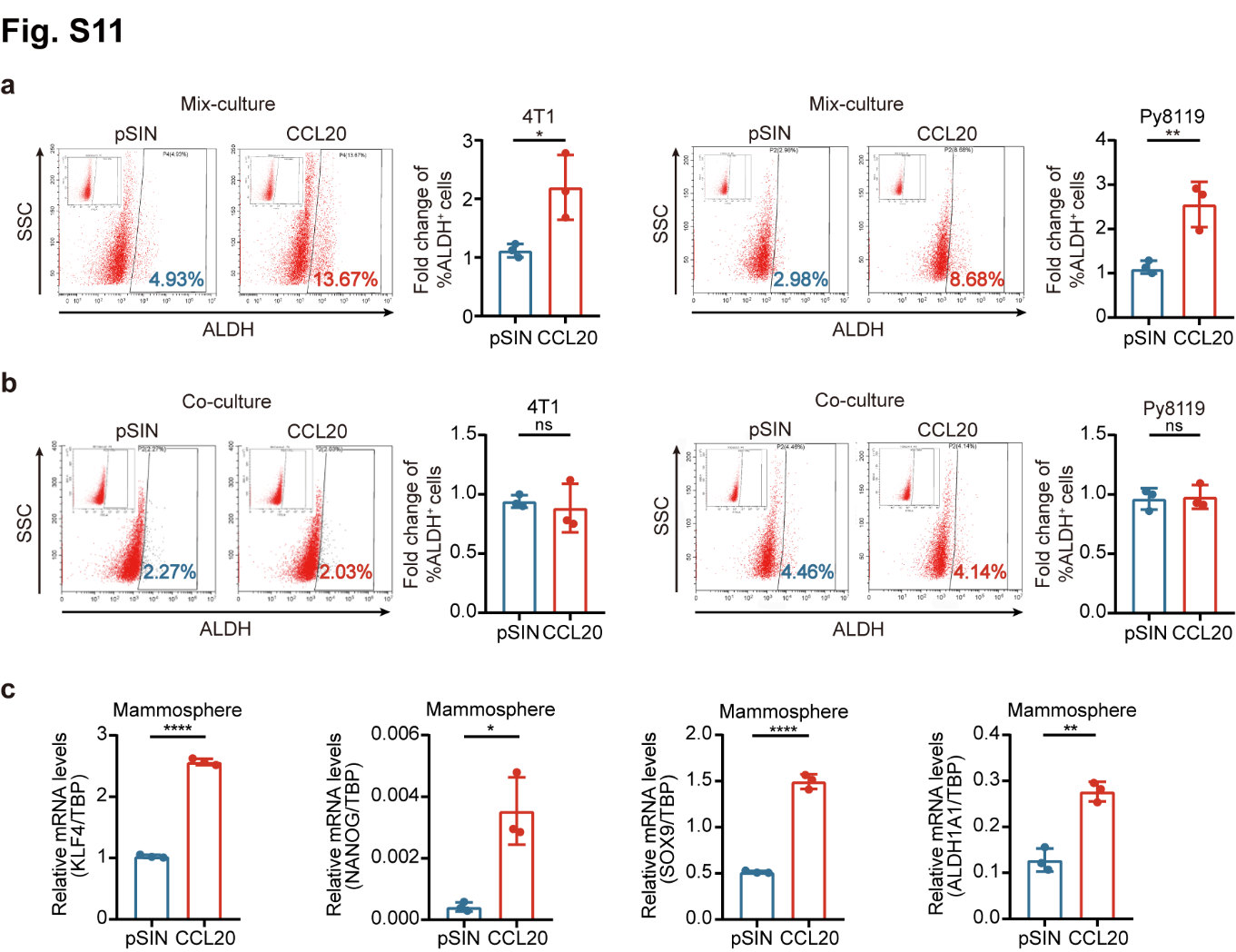


Fig. S11. CCL20-modulated PMN-MDSCs enhanced the stemness of breast cancer cells.

Balb/c mice were orthotopically transplanted with pSIN-/CCL20-overexpressing 4T1 cells (5×10^4^) at the fourth mammary fat pads (*n*=5 for each group). C57BL/6N mice were orthotopically transplanted with pSIN-/CCL20-overexpressing Py8119 cells (1×10^5^) at the fourth mammary fat pads (*n*=5 for each group). **a** 4T1 or Py8119 cells were mix-cultured with pSIN-/CCL20-modulated PMN-MDSCs for 3 days. The percentage of ALDH^+^ BCSCs was determined by the ALDEFLUOR assay in 4T1 (left) or Py8119 (right) cells. Bar graph was presented as the mean of three biologically independent experiments (mean ± SEM). **b** M-MDSCs were sorted from pSIN-/CCL20-overexpressing 4T1 or Py8119 cell allografts, and then co-cultured with 4T1 or Py8119 cells for 3 days, respectively. The percentage of ALDH^+^ BCSCs was determined by the ALDEFLUOR assay in 4T1 (left) or Py8119 (right) cells. Bar graph was presented as the mean of three biologically independent experiments (mean ± SEM). **c** Primary mammosphere formation in 4T1 cells mix-cultured with pSIN-/CCL20-modulated PMN-MDSCs. The mRNA expression levels of several stemness-related genes in 4T1 cells were analyzed by qRT-PCR. Bar graph was presented as the mean of three biologically independent experiments (mean ± SEM). ns, no significance; **p* < 0.05, ***p* < 0.01, *****p* < 0.0001.


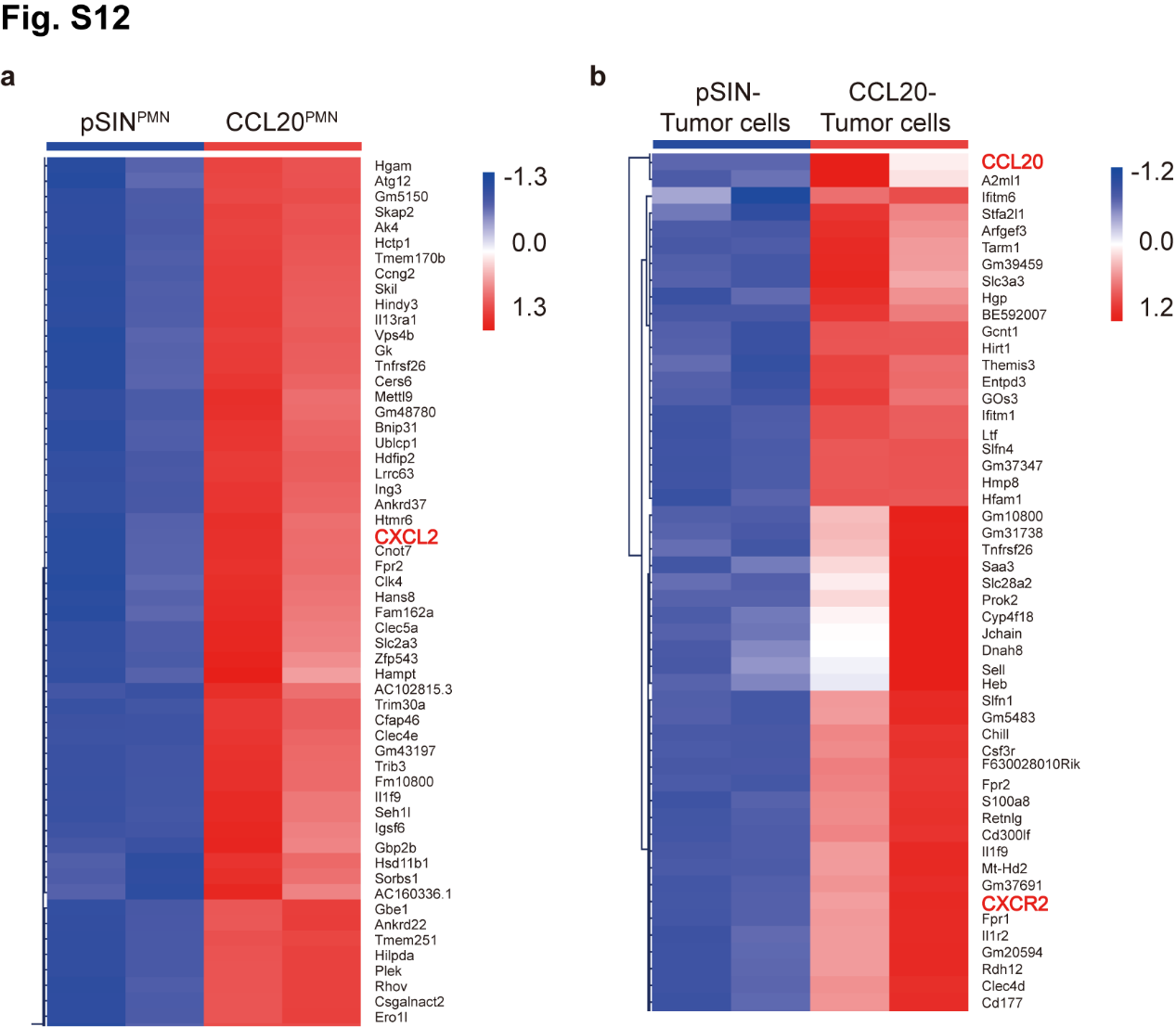


Fig. S12. CXCL2 and CXCR2 were upregulated in PMN-MDSCs and tumor cells in CCL20-overexpressing 4T1 cell allograft tumors respectively.

**a, b** Top fifty up-regulated genes in PMN-MDSCs (a) or tumor cells (b) from pSIN-/CCL20-overexpressing 4T1 cell allograft tumors were shown in the heatmap.


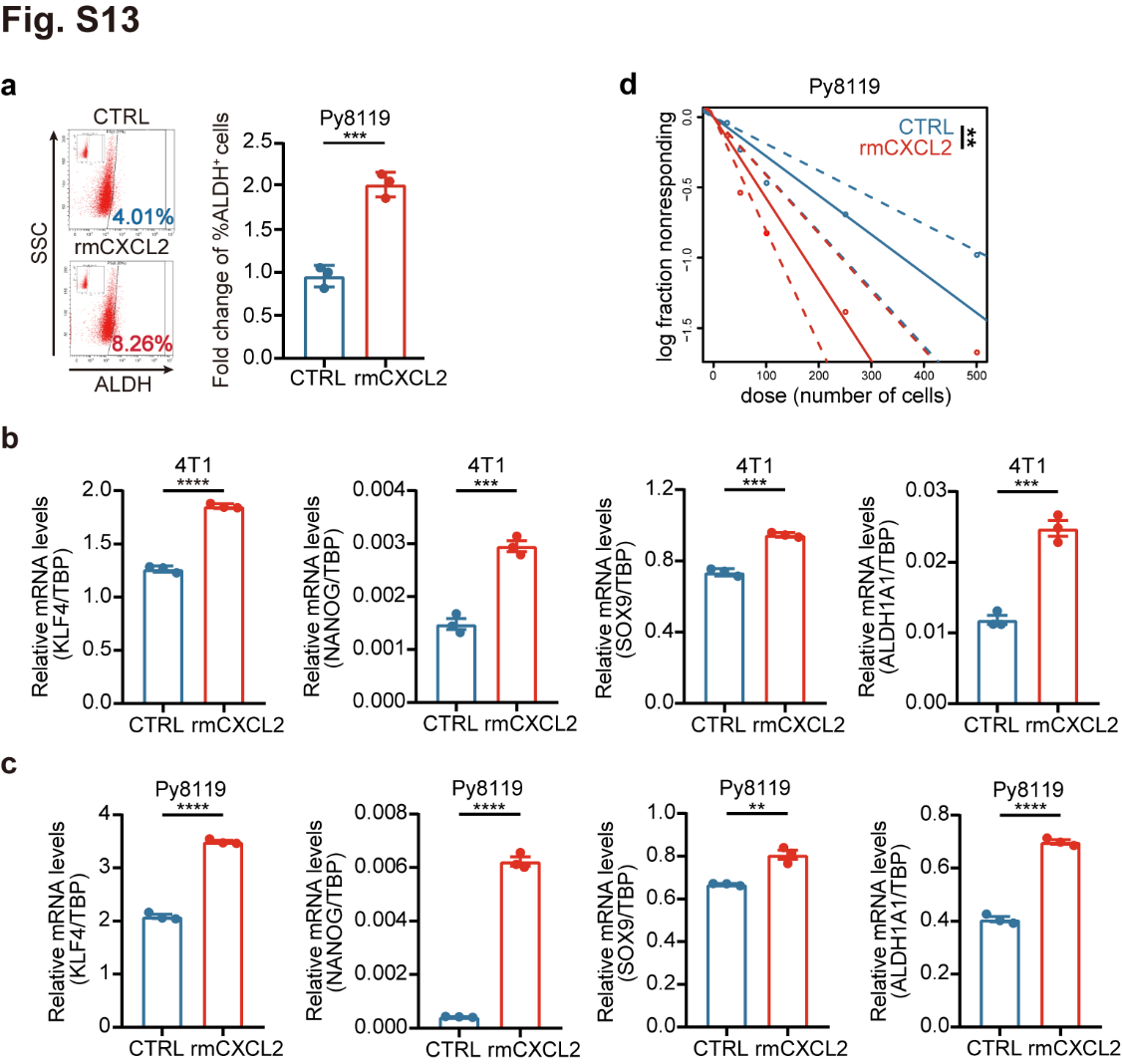


Fig. S13. rmCXCL2 significantly increased the stemness of breast cancer cells.

**a** Py8119 cells were treated with rmCXCL2 (10 ng/ml) for 3 days, and the percentage of ALDH^+^ BCSCs was determined by the ALDEFLUOR assay. Bar graph was presented as the mean of three biological independent experiments (mean ± SEM). **b, c** 4T1 or Py8119 cells were treated with rmCXCL2 (10 ng/ml) for 3 days, and the mRNA expression levels of several stemness-related genes in 4T1 (b) or Py8119 cells (c) were analyzed by qRT-PCR. **d** *In vitro* LDA of Py8119 cells treated with rmCXCL2 (10 ng/ml) for 3 days. ***p* < 0.01, ****p* < 0.001, *****p* < 0.0001.


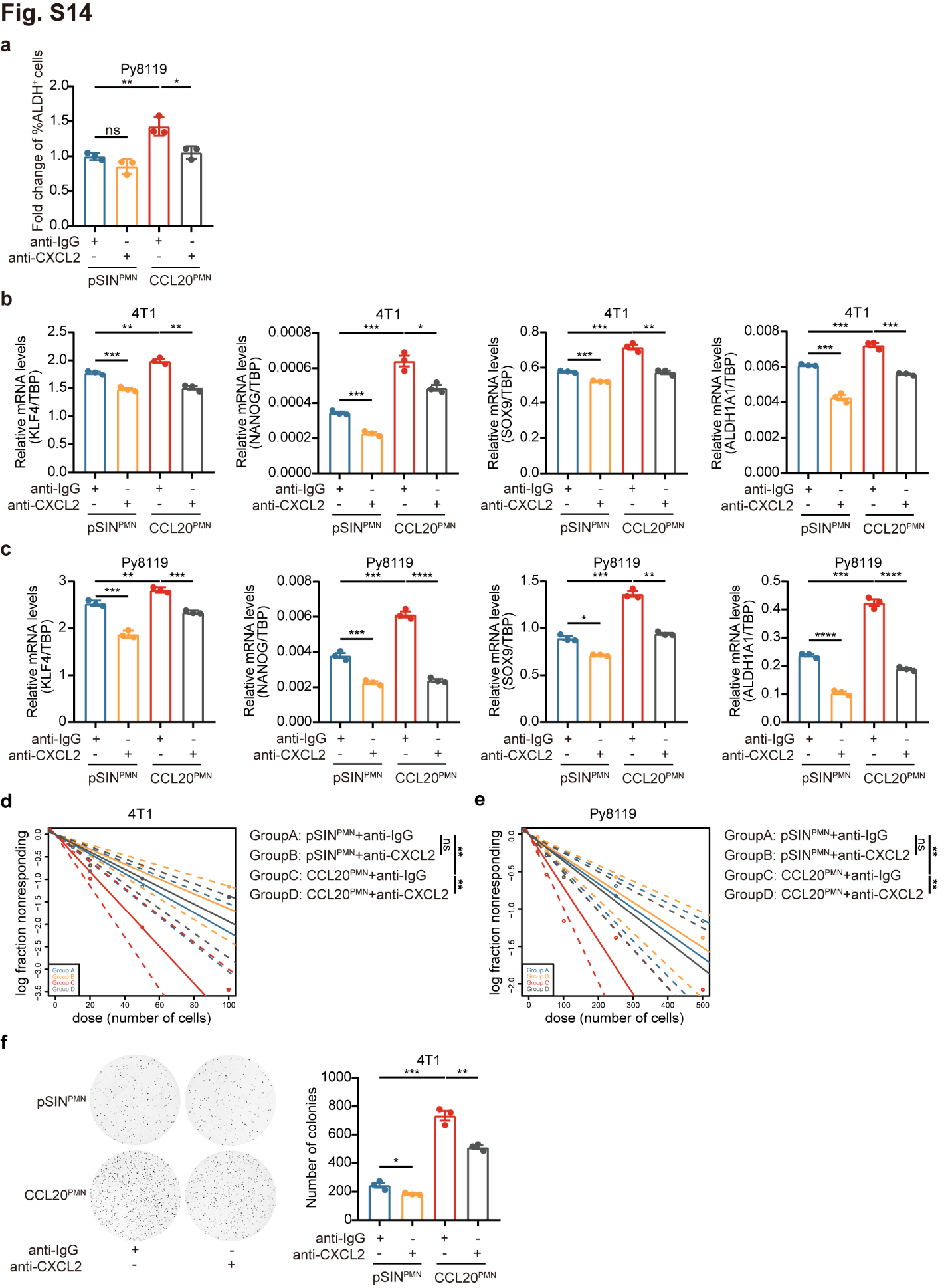


Fig. S14. CXCL2 secreted by CCL20-modulated PMN-MDSCs increased the stemness of breast cancer cells.

**a** Py8119 cells were co-cultured with pSIN^PMN^ or CCL20^PMN^ from pSIN-/CCL20-overexpressing py8119 cell allograft tumors. Then, the CXCL2 neutralizing antibody (anti-CXCL2, 2 µg/ml) or control (anti-IgG, 2 µg/ml) was administrated for 3 days, and the percentage of ALDH^+^ BCSCs was determined by the ALDEFLUOR assay in Py8119 cells. Bar graph was presented as the mean of three biological independent experiments (mean ± SEM). **b-e** 4T1 or Py8119 cells were co-cultured with pSIN^PMN^ or CCL20^PMN^ from pSIN-/CCL20-overexpressing 4T1 or Py8119 cell allograft tumors. Then, the CXCL2 neutralizing antibody (anti-CXCL2, 2 µg/ml) or control (anti-IgG, 2 µg/ml) was administrated for 3 days. The mRNA expression levels of several stemness-related genes in 4T1 (b) or Py8119 cells (c) were analyzed by qRT-PCR. *In vitro* LDA was performed with 4T1 (d) or Py8119 cells (e). **f** 4T1 cells were co-cultured with pSIN^PMN^ or CCL20^PMN^ from pSIN-/CCL20-overexpressing 4T1 cell allograft tumors. Then, the CXCL2 neutralizing antibody (anti-CXCL2, 2 µg/ml) or control (anti-IgG, 2 µg/ml) was administrated for 3 days. Soft agar colony formation assay was performed with 4T1 cells after the pretreatment. The colony images were taken (left) and the colony numbers were quantified (right). Bar graph was presented as the mean of three biologically independent experiments (mean ± SEM). **p* < 0.05, ***p* < 0.01, ****p* < 0.001, *****p* < 0.0001.

**
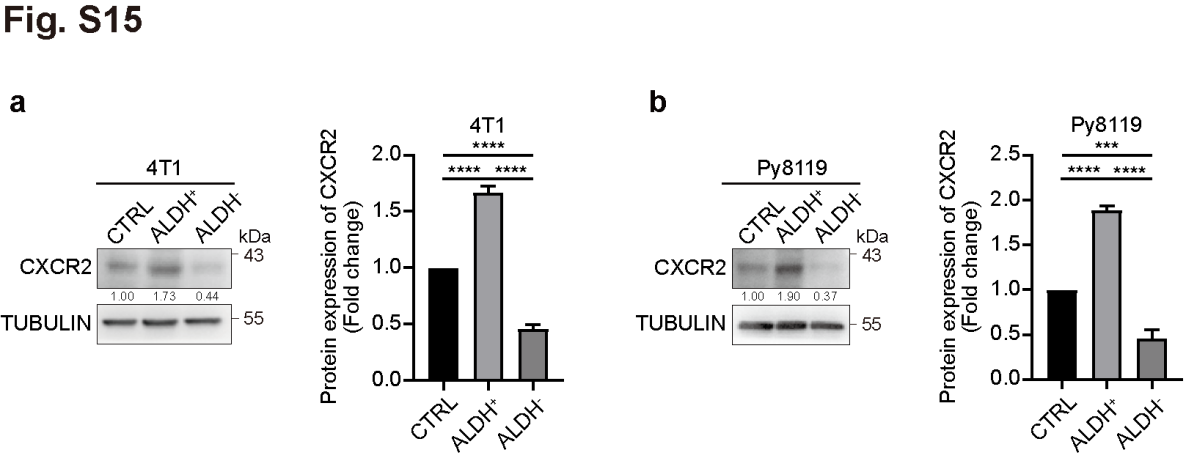
**

Fig. S15. CXCR2 was highly expressed in ALDH^+^ BCSCs.

**a, b** 4T1 (a) or Py8119 (b) cells were sorted into ALDH^+^ and ALDH^-^ cells by flow cytometry. The protein expression levels of CXCR2 in total (CTRL), ALDH^+^ and ALDH^-^ cells were analyzed by western blotting. TUBULIN was utilized as the internal control. Bar graph was presented as the mean of three biologically independent experiments (mean ± SEM). ****p* < 0.001. *****p* < 0.0001.


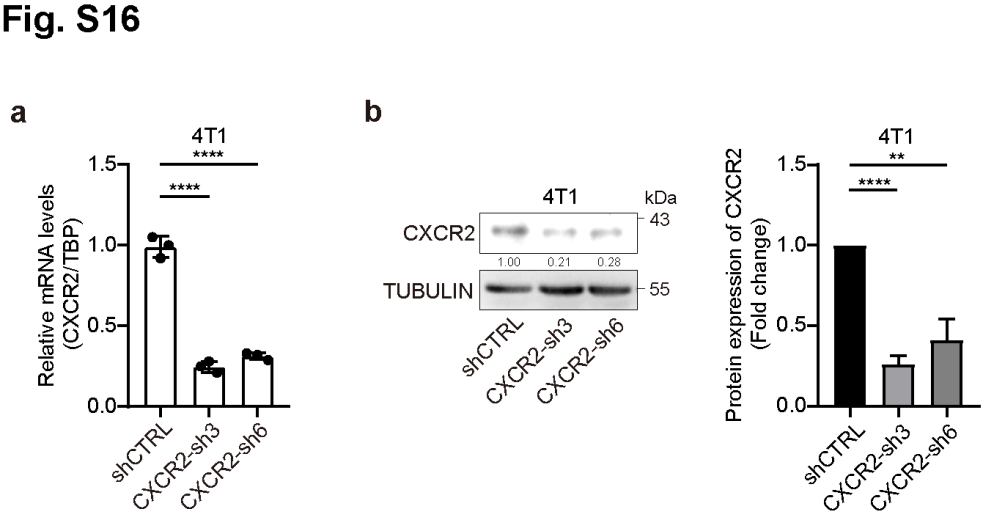


Fig. S16. The establishment of CXCR2-knockdown 4T1 cells.

**a, b** The knockdown efficiency of CXCR2 in 4T1 cells was analyzed by both qRT-PCR (a) and western blotting (b). TUBULIN was utilized as the internal control. Bar graph was presented as the mean of three biologically independent experiments (mean ± SEM). ***p* < 0.01. *****p* < 0.0001.

**
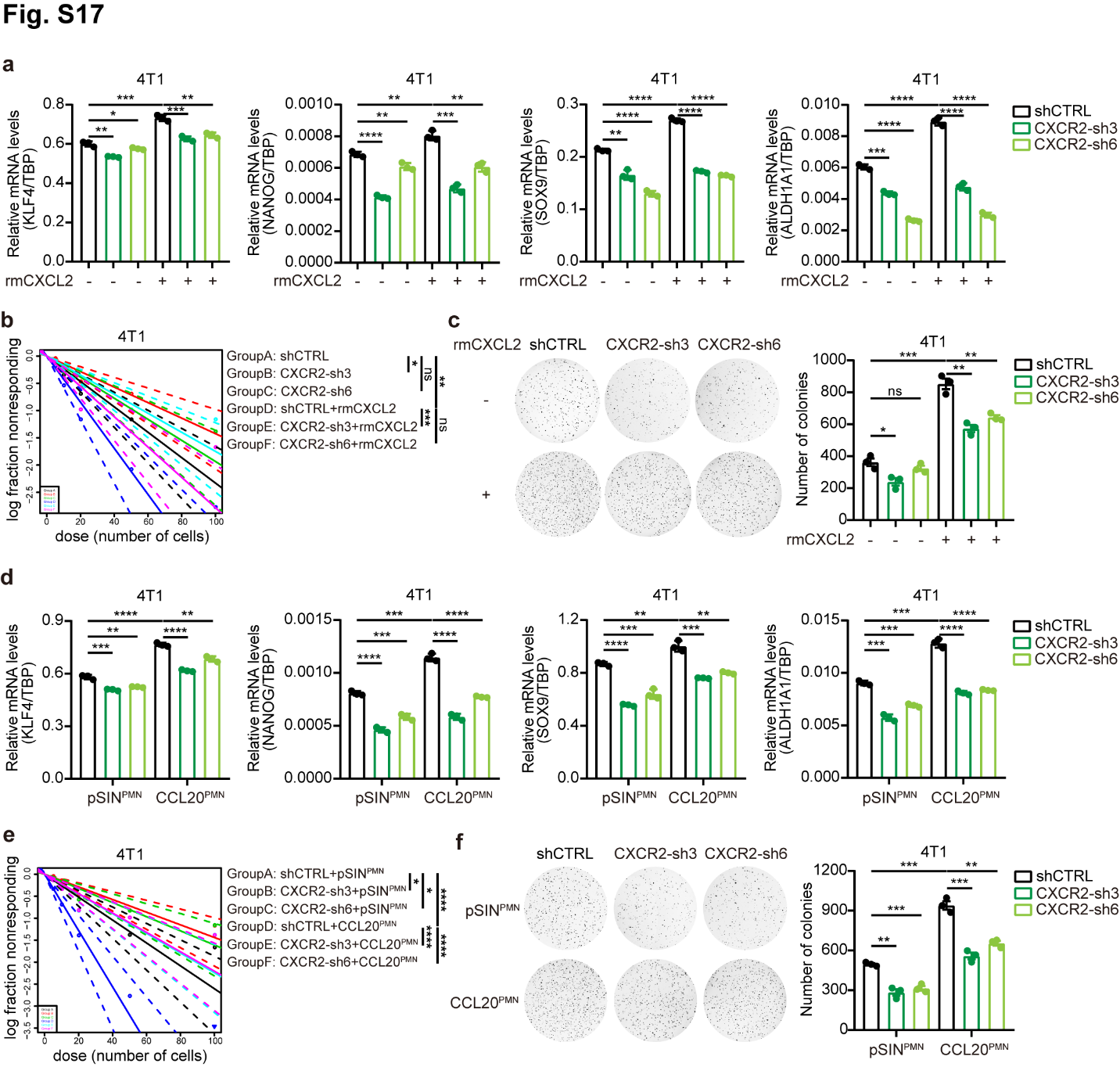
**

Fig. S17. CXCR2 knockdown significantly inhibited the increase of breast cancer cell stemness induced by CXCL2 from CCL20-modulated PMN-MDSCs.

**a-c** 4T1 scramble (shCTRL) or CXCR2-knockdown cells (CXCR2-sh3 and CXCR2-sh6) were treated with rmCXCL2 (10 ng/ml) for 3 days. The mRNA expression levels of several stemness-related genes were analyzed by qRT-PCR (a). *In vitro* LDA was performed (b). Soft agar colony formation assay was performed, the colony images were taken (left) and the colony numbers were quantified (right) (c). Bar graph was presented as the mean of three biologically independent experiments (mean ± SEM). **d-f** 4T1 scramble (shCTRL) or CXCR2-knockdown cells (CXCR2-sh3 and CXCR2-sh6) were co-cultured with pSIN^PMN^ or CCL20^PMN^ sorted from pSIN-/CCL20-overexpressing 4T1 cell allograft tumors. The mRNA expression levels of several stemness-related genes were analyzed by qRT-PCR (d). *In vitro* LDA was performed (e). Soft agar colony formation assay was performed, the colony images were taken (left) and the colony numbers were quantified (right) (f). Bar graph was presented as the mean of three biologically independent experiments (mean ± SEM). ns, no significance; **p* < 0.05, ***p* < 0.01, ****p* < 0.001, *****p* < 0.0001.

**
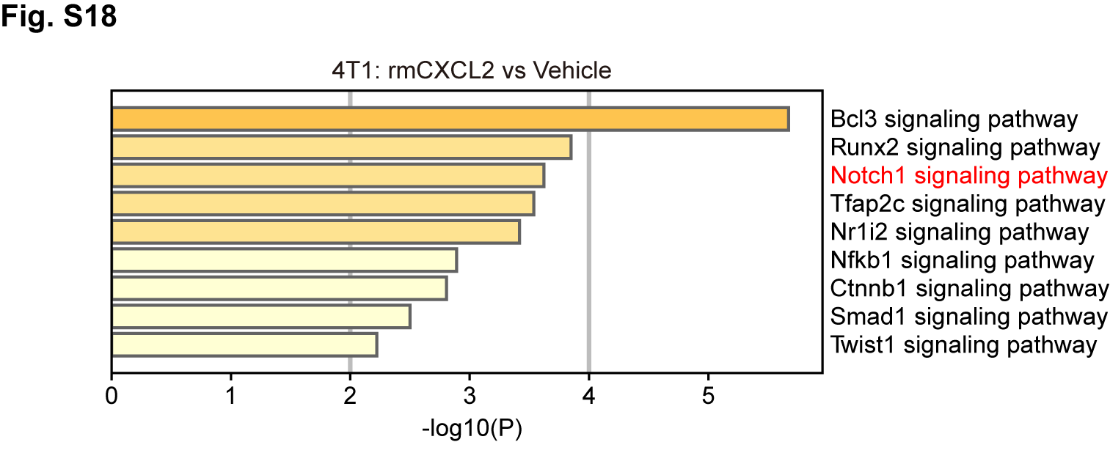
**

Fig. S18. CXCL2 activated the NOTCH1 signaling pathway.

Metascape analysis (https://metascape.org/gp/index.html#/main/step1) showed NOTCH1-related genes were enriched in 4T1 cells treated with rmCXCL2 (10 ng/ml) compared to PBS.


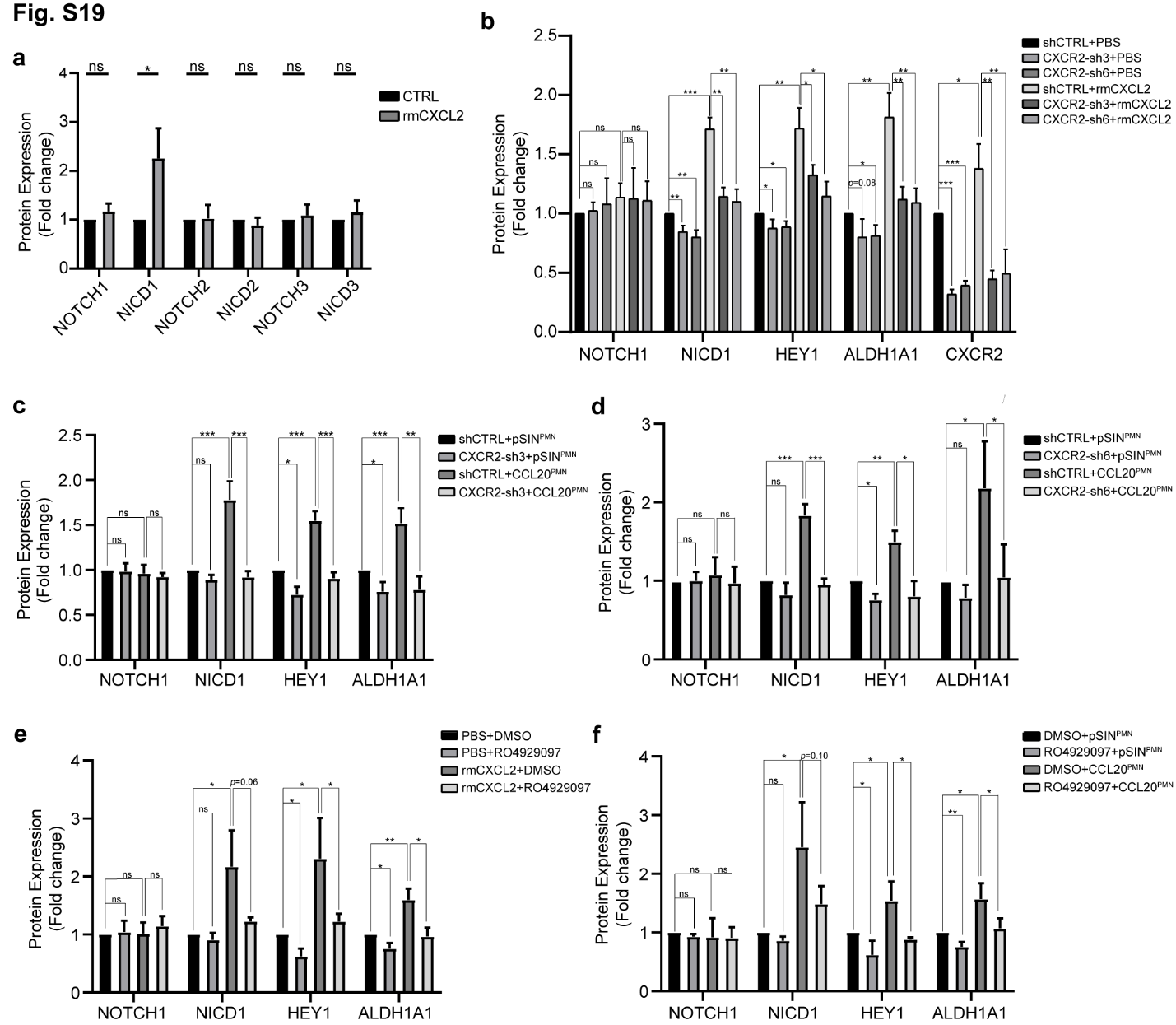


Fig. S19. CXCL2-CXCR2 axis enhanced the stemness of breast cancer cells through activating NOTCH1/HEY1 pathway.

**a** The protein expression levels of NOTCH1/2/3 and NICD1/2/3 in 4T1 cells treated with rmCXCL2 (10 ng/ml) or PBS for 3 days were analyzed by western blotting. TUBULIN was utilized as the internal control. Bar graph was presented as the mean of three biological independent experiments (mean ± SEM). **b** Cells were treated with rmCXCL2 (10 ng/ml) or PBS for 3 days, and the protein levels were analyzed by western blotting. TUBULIN was utilized as the internal control. Bar graph was presented as the mean of three biologically independent experiments (mean ± SEM). **c, d** PMN-MDSCs were sorted from pSIN-/CCL20-overexpressing 4T1 cell allograft tumors (pSIN^PMN^/CCL20^PMN^). 4T1 scramble (shCTRL) or CXCR2-knockdown cells (CXCR2-sh3, c; CXCR2-sh6, d) were co-cultured with pSIN^PMN^ or CCL20^PMN^ for 3 days. The protein expression levels of NOTCH1, NICD1, HEY1 and ALDH1A1 in 4T1 cells were analyzed by western blotting. TUBULIN was utilized as the internal control. Bar graph was presented as the mean of three biologically independent experiments (mean ± SEM). **e** 4T1 cells were treated with rmCXCL2 (10 ng/ml) or/and NOTCH inhibitor RO4929097 (1 µM) for 3 days. The protein expression levels in 4T1 cells were analyzed by western blotting. TUBULIN was utilized as the internal control. Bar graph was presented as the mean of three biologically independent experiments (mean ± SEM). **f** 4T1 cells were co-cultured with pSIN^PMN^ or CCL20^PMN^ and simultaneously treated with RO4929097 (1 µM) or DMSO for 3 days. The protein levels of NOTCH1, NICD1, HEY1 and ALDH1A1 in 4T1 cells were analyzed by western blotting. TUBULIN was utilized as the internal control. Bar graph was presented as the mean of three biologically independent experiments (mean ± SEM). ns, no significance; **p* < 0.05, ***p* < 0.01, ****p* < 0.001.


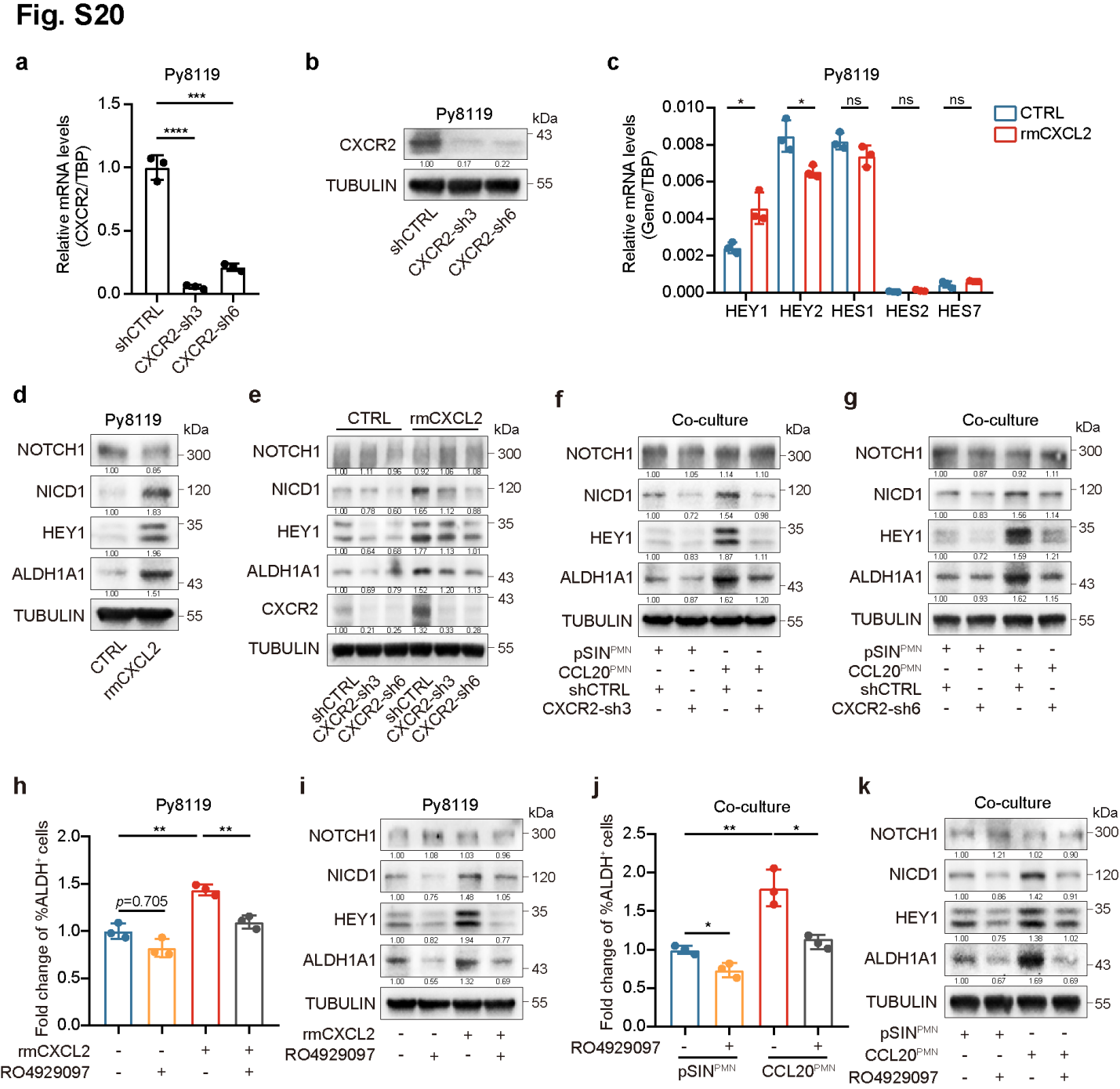


Fig. S20. CXCL2-CXCR2 axis enhanced the stemness of breast cancer cells through activating NOTCH1/HEY1 pathway.

**a, b** CXCR2 knockdown cells were established in Py8119 cells. The knockdown efficiency of CXCR2 in Py8119 cells was analyzed by both qRT-PCR (a) and western blotting (b). Bar graph was presented as the mean of three biologically independent experiments (mean ± SEM). **c** The mRNA expression levels of *HEY1*, *HEY2*, *HES1*, *HES2* and *HES7* in Py8119 cells treated with rmCXCL2 (10 ng/ml) or PBS for 3 days were analyzed by qRT-PCR. Bar graph was presented as the mean of three biological independent experiments (mean ± SEM). **d** The protein levels of NOTCH1, NICD1, HEY1 and ALDH1A1 in Py8119 cells treated with rmCXCL2 (10 ng/ml) or PBS for 3 days were analyzed by western blotting. **e** Cells were treated with rmCXCL2 (10 ng/ml) or PBS for 3 days, and the protein levels were analyzed by western blotting. **f, g** PMN-MDSCs were sorted from pSIN-/CCL20-overexpressing Py8119 cell allograft tumors (pSIN^PMN^/CCL20^PMN^). Py8119 scramble (shCTRL) or CXCR2-knockdown cells (CXCR2-sh3, f; CXCR2-sh6, g) were co-cultured with pSIN^PMN^ or CCL20^PMN^ for 3 days. The protein expression levels of NOTCH1, NICD1, HEY1 and ALDH1A1 in Py8119 cells were analyzed by western blotting. **h, i** Py8119 cells were treated with rmCXCL2 (10 ng/ml) or/and NOTCH inhibitor RO4929097 (1 µM) for 3 days. The percentage of ALDH^+^ BCSCs in Py8119 cells was determined by ALDEFLUOR assay and bar graph was shown as mean ± SEM (h). The protein expression levels in Py8119 cells were analyzed by western blotting (i). **j, k** Py8119 cells were co-cultured with pSIN^PMN^ or CCL20^PMN^ and simultaneously treated with RO4929097 (1 µM) or DMSO for 3 days. The percentage of ALDH^+^ BCSCs in Py8119 cells was determined by ALDEFLUOR assay (j), and the protein levels of NOTCH1, NICD1, HEY1 and ALDH1A1 in Py8119 cells were analyzed by western blotting (k). Bar graph was presented as the mean of three biological independent experiments (mean ± SEM). ns, no significance; **p* < 0.05, ***p* < 0.01, ****p* < 0.001, *****p* < 0.0001.


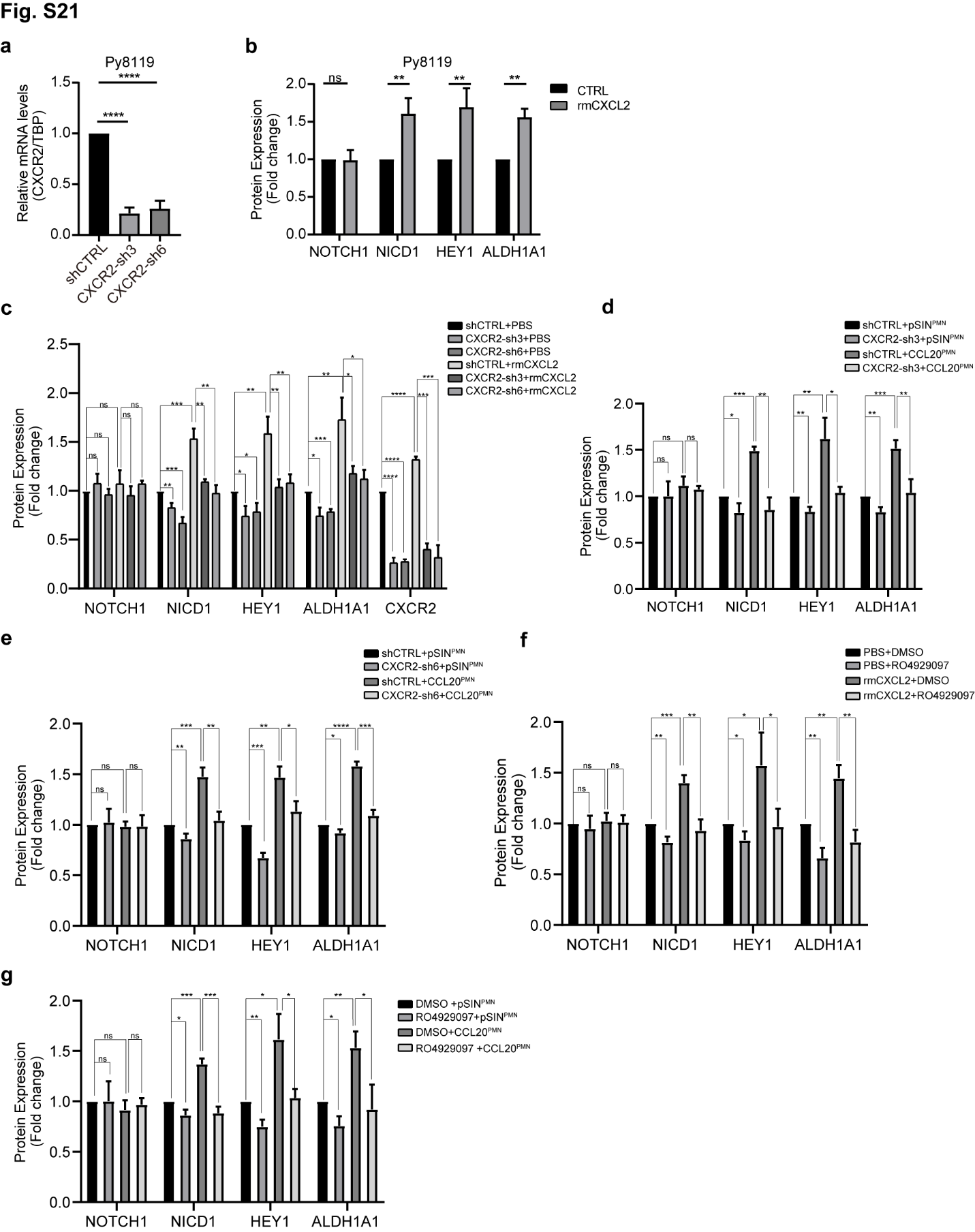
Fig. S21. CXCL2-CXCR2 axis promoted the stemness of breast cancer cells by activating NOTCH1/HEY1 pathway.

**a** CXCR2 knockdown cells were established in Py8119 cells. The knockdown efficiency of CXCR2 in py8119 cells was analyzed by western blotting. TUBULIN was utilized as the internal control. Bar graph was presented as the mean of three biological independent experiments (mean ± SEM). **b** The protein levels of NOTCH1, NICD1, HEY1 and ALDH1A1 in Py8119 cells treated with rmCXCL2 (10 ng/ml) or PBS for 3 days were analyzed by western blotting. TUBULIN was utilized as the internal control. Bar graph was presented as the mean of three biological independent experiments (mean ± SEM). **c** Cells were treated with rmCXCL2 (10 ng/ml) or PBS for 3 days, and the protein levels were analyzed by western blotting. TUBULIN was utilized as the internal control. Bar graph was presented as the mean of three biological independent experiments (mean ± SEM). **d, e** PMN-MDSCs were sorted from pSIN-/CCL20-overexpressing Py8119 cell allograft tumors (pSIN^PMN^/CCL20^PMN^). Py8119 scramble (shCTRL) or CXCR2-knockdown cells (CXCR2-sh3, d; CXCR2-sh6, e) were co-cultured with pSIN^PMN^ or CCL20^PMN^ for 3 days. The protein expression levels of NOTCH1, NICD1, HEY1 and ALDH1A1 in Py8119 cells were analyzed by western blotting. TUBULIN was utilized as the internal control. Bar graph was presented as the mean of three biological independent experiments (mean ± SEM). **f** Py8119 cells were treated with rmCXCL2 (10 ng/ml) or/and NOTCH inhibitor RO4929097 (1 µM) for 3 days. The protein expression levels in Py8119 cells were analyzed by western blotting. TUBULIN was utilized as the internal control. Bar graph was presented as the mean of three biological independent experiments (mean ± SEM). **g** Py8119 cells were co-cultured with pSIN^PMN^ or CCL20^PMN^ and simultaneously treated with RO4929097 (1 µM) or DMSO for 3 days. The protein levels of NOTCH1, NICD1, HEY1 and ALDH1A1 in Py8119 cells were also analyzed by western blotting. TUBULIN was utilized as the internal control. Bar graph was presented as the mean of three biological independent experiments (mean ± SEM). ns, no significance; **p* < 0.05, ***p* < 0.01, ****p* < 0.001, *****p* < 0.0001.


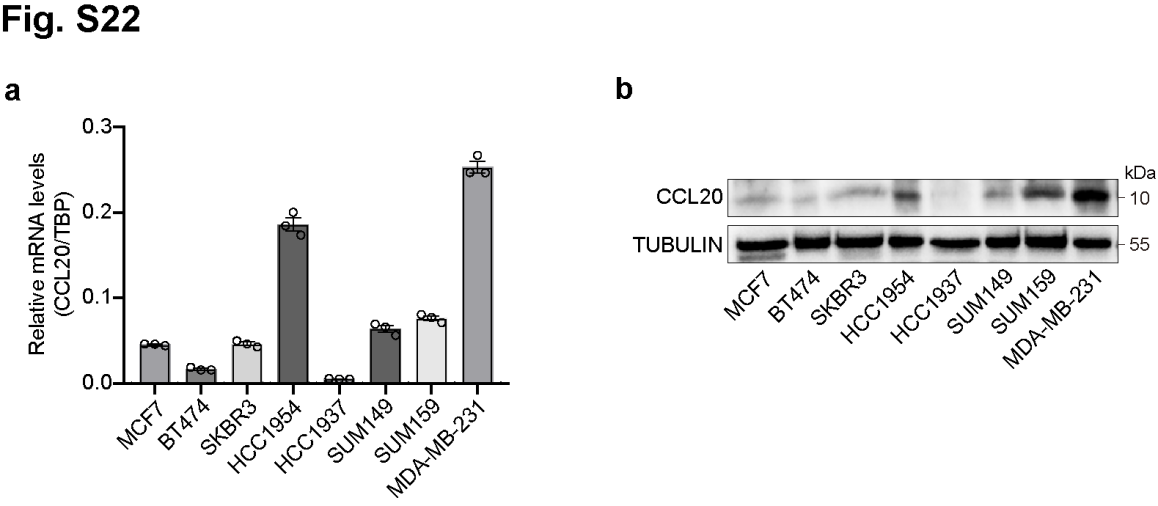


Fig. S22. MDA-MB-231 cells showed higher CCL20 expression.

**a, b** The mRNA or protein expression levels of CCL20 in a series of human breast cancer cell lines were analyzed by both qRT-PCR (a) and western blotting (b).

**
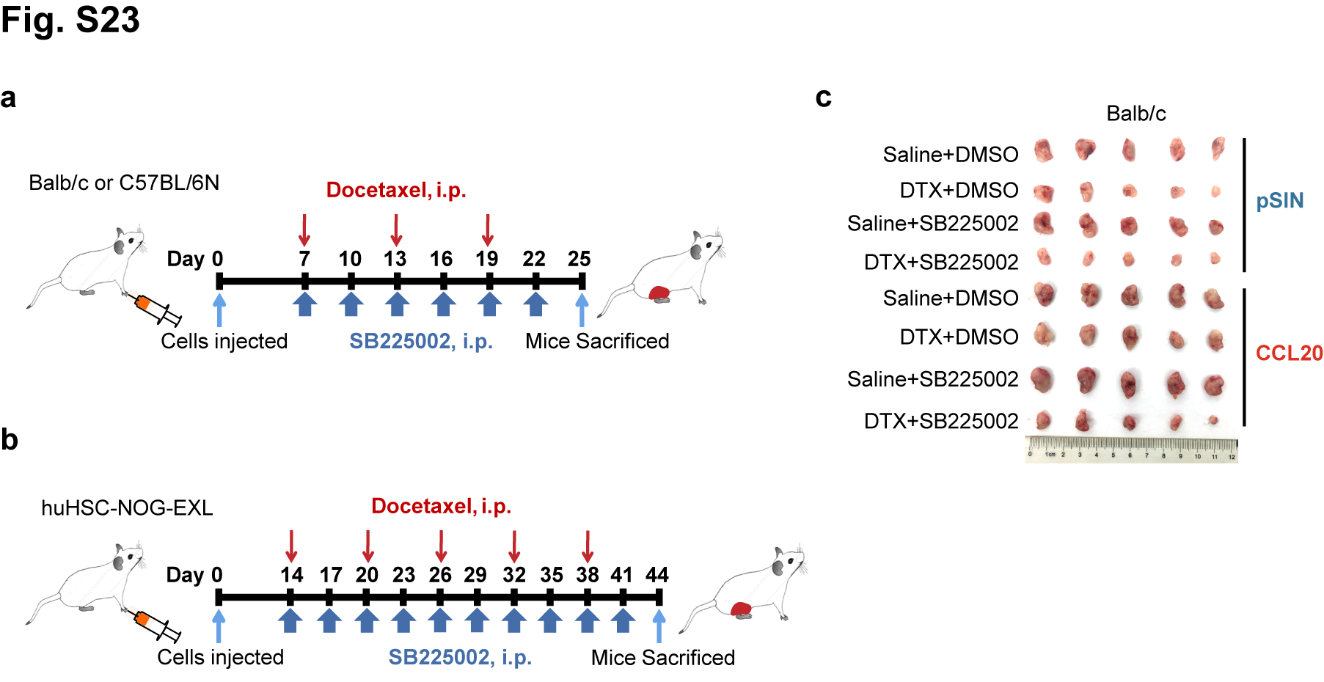
**

Fig. S23. The combinational treatment strategy by combining DTX with CXCR2 antagonist SB225002 in orthotopic breast tumor mouse models.

**a** The experimental diagram for the combinational treatment strategy. Balb/c or C57BL/6N mice were orthotopically transplanted with pSIN-/CCL20-overexpressing 4T1 cells (2×10^4^) or Py8119 cell (1×10^5^) at the fourth mammary fat pads (*n*=5 for each group). After 7 days, mice bearing palpable tumors were treated with vehicle control, docetaxel (DTX, 20 mg/kg for Balb/c mice, 40 mg/kg for C57BL/6N mice, i.p., once every six days) alone, CXCR2 antagonist SB225002 (10 mg/kg, i.p., once every three days) alone or in combination. **b** The experimental diagram for the combinational treatment strategy. huHSC-NOG-EXL mice were orthotopically transplanted with MDA-MB-231 cells (2×10^6^) at the fourth mammary fat pads (*n*=4 for each group). After 14 days, mice bearing tumors were treated with vehicle control, docetaxel (DTX, 20 mg/kg, i.p., once every six days) alone, CXCR2 antagonist SB225002 (10 mg/kg, i.p., once every three days) alone or in combination. **c** Tumor images of Balb/c mice were taken after mice were sacrificed.

**
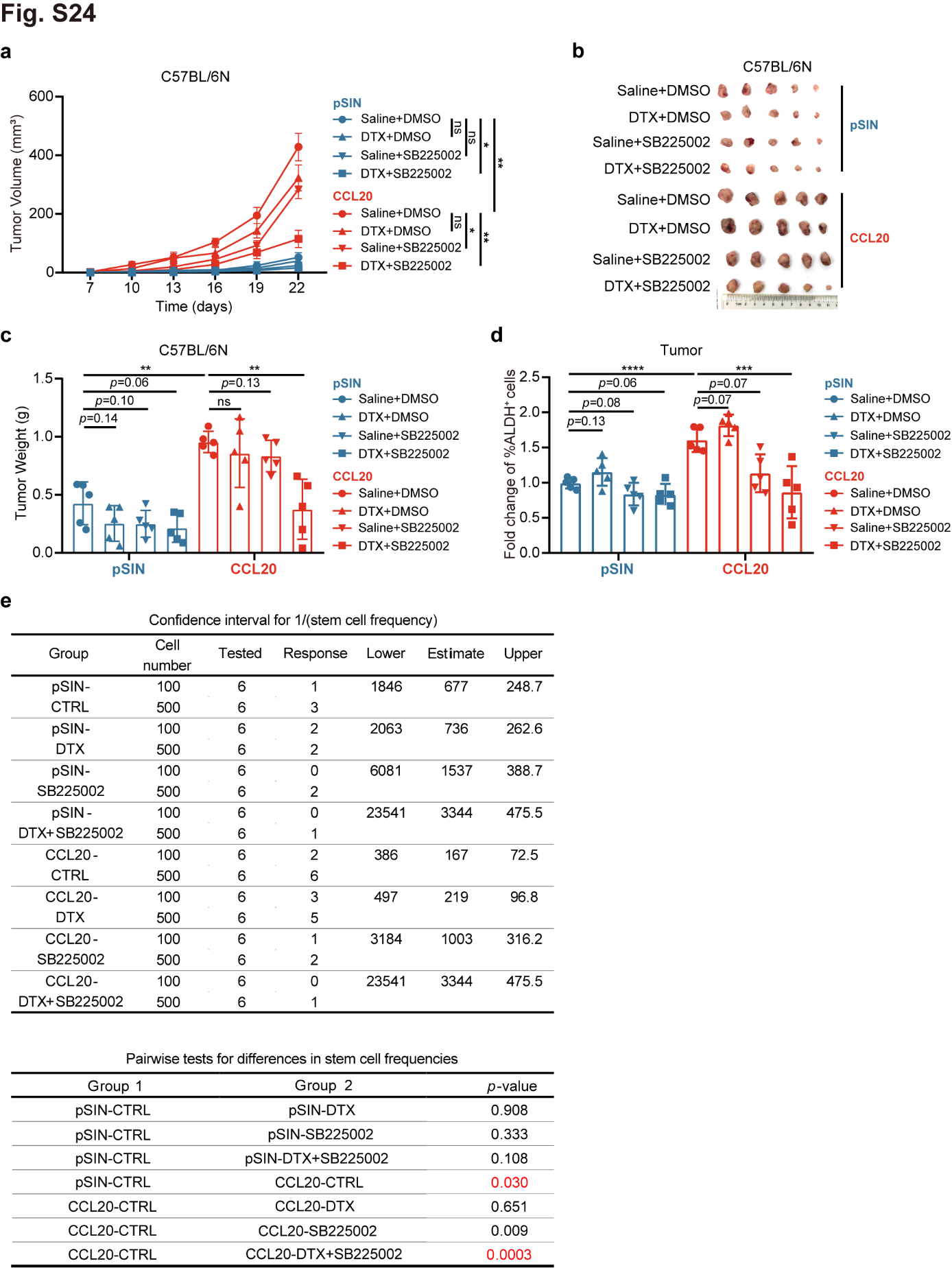
**

Fig. S24. CXCR2 antagonist enhanced the therapeutic efficacy of docetaxel on breast tumors in C57BL/6N mice.

C57BL/6N mice were orthotopically transplanted with pSIN-/CCL20-overexpressing Py8119 cells (1×10^5^) at the fourth mammary fat pads (*n*=5 for each group). After 7 days, mice bearing palpable tumors were treated with vehicle control, DTX (40 mg/kg, i.p., once every six days) alone, SB225002 (10 mg/kg, i.p., once every three days) alone or in combination. After 25 days, mice were sacrificed. **a-c** Tumor size was monitored every 3 days and tumor volume was calculated (a). Tumor image (b) and tumor weight (c) were shown. **d** The percentage of ALDH^+^ BCSCs was determined by ALDEFLUOR assay in tumor cells from Py8119 cell allograft tumors and shown in bar graph as mean ± SEM. **e** Tumor cells (CD45^-^CD140b^-^CD31^-^) isolated from SB225002- and/or DTX-treated tumors were engrafted to mammary fat pads of C57BL/6N mice at a limited dilution (*n*=3 for each group, two sites each mouse, 100 or 500 cells/site). The stem cell frequency and *p-*value calculation were based on the positive tumor sites. ns, no significance; **p* < 0.05; ***p* < 0.01.


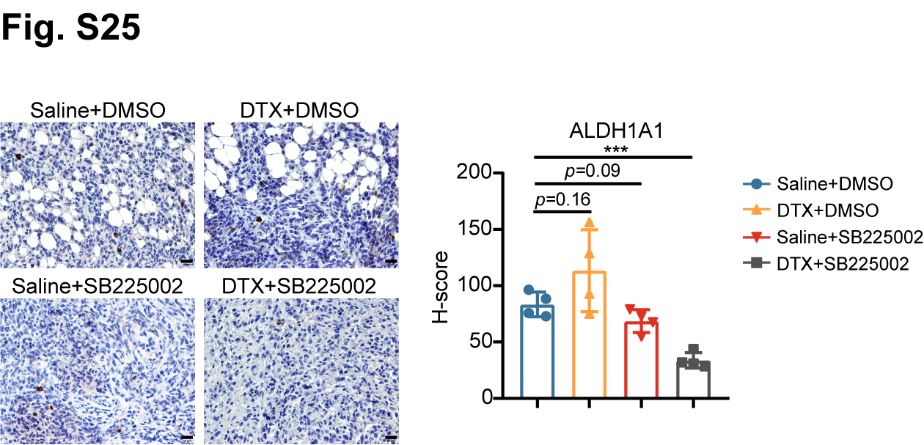


Fig. S25. The combinational treatment effectively decreased ALDH^+^ BCSCs in humanized mice bearing CCL20^high^-expressing breast cancer cell allograft tumors.

ALDH1A1 expression in tumor tissues of huHSC-NOG-EXL mice was analyzed by IHC staining. Representative images (left) and bar graph of H Scores (right, mean ± SEM) were shown. Scale bar, 20 μm. ns, no significance; ****p* < 0.001.


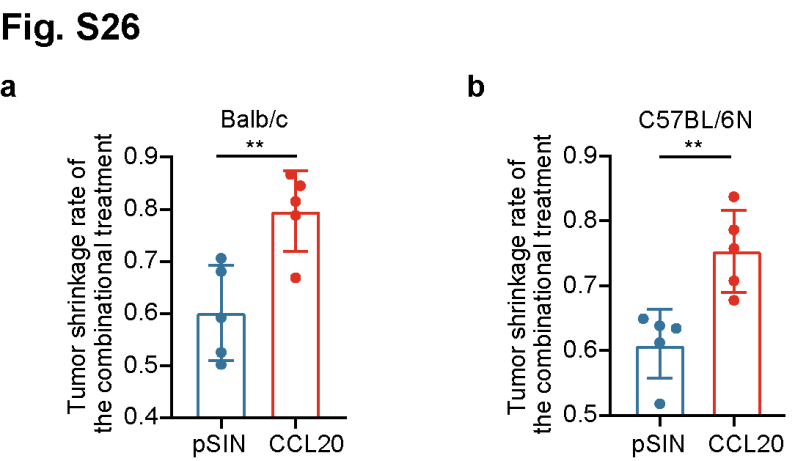


Fig. S26. The percentage of tumor shrinkage was higher in CCL20-overexpressing group than in pSIN group under the combinational treatment.

**a, b** The percentage of tumor shrinkage in Balb/c (a) or C57BL/6N (b) mice bearing breast cancer cell orthotopic allograft tumors was analyzed in CCL20-overexpressing group compared to pSIN group under the combinational treatment. Bar graph were shown as mean ± SEM. ***p* < 0.01.


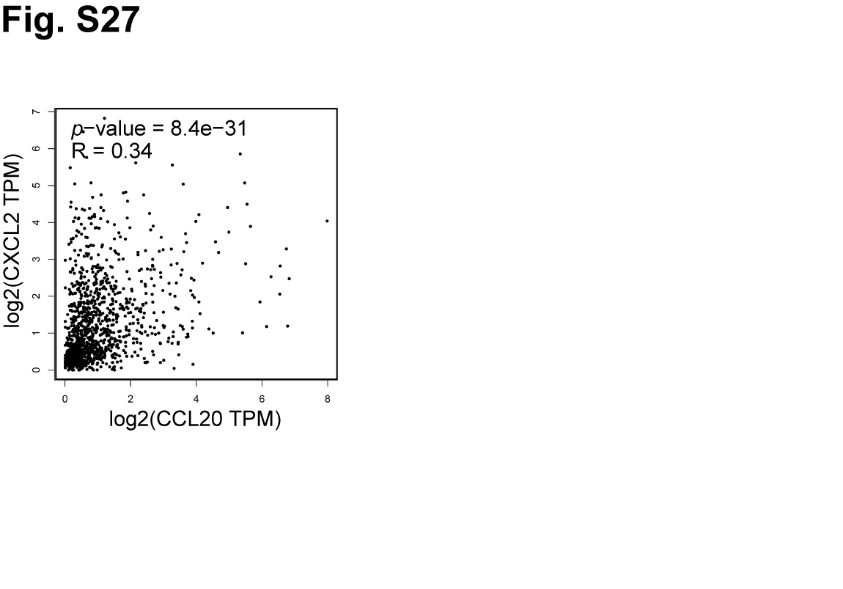


Fig. S27. The correlation between CCL20 expression and CXCL2 expression in patients’ breast tumors.

CCL20 mRNA expression was positively correlated with the mRNA expression of CXCL2 in GEPIA2 database (http://gepia2.cancer-pku.cn/#index).

Table S1. Primer sequences for plasmid construction

| Target | Sequence (5'→3') |
| --- | --- |
| shCXCR2-3 | GATTCTCGAGAATCTCCGTAGCATTCAAGGCGGTGTTTCGTCCTTTCC |
| shCXCR2-6 | CATACTCGAGTATGAGAATATCTTGCACAGGCGGTGTTTCGTCCTTTCC |

Table S2. Primer sequence information for qRT-PCR

| Target | Forward (5'→3') | Reverse (3'→5') |
| --- | --- | --- |
| mCCL20 | CCAGGCAGAAGCAAGCAACT | TCGGCCATCTGTCTTGTGAA |
| huCCL20 | TGCTGTACCAAGAGTTTGCTC | CGCACACAGACAACTTTTTCTTT |
| mNANOG | TCTTCCTGGTCCCCACAGTTT | GCAAGAATAGTTCTCGGGATGAA |
| mKLF4 | AACTACCCTCCTTTCCTGCC | GACCTTCTTCCCCTCTTTGG |
| mSOX9 | GACTCCCCACATTCCTCCTC | CCCTCTCGCTTCAGATCAAC |
| mALDH1A1 | AGCCCACAGTGTTCTCCAAC | CCTGCAGAGCAGATGACACA |
| mCXCL2 | GCCAAGGGTTGACTTCAAGA | TTCAGGGTCAAGGCAAACTT |
| mCXCR2 | AGCCACTCTGCTCACAAACA | AGGGCATGCCAGAGCTATAA |
| mHEY1 | GCGCGGACGAGAATGGAAA | TCAGGTGATCCACAGTCATCTG |
| mHEY2 | AAGCGCCCTTGTGAGGAAAC | GGTAGTTGTCGGTGAATTGGAC |
| mHES1 | TCAACACGACACCGGACAAAC | ATGCCGGGAGCTATCTTTCTT |
| mHES2 | CTGAAGGGTCTCGTATTGCCG | CGCAGGTGCTCTAGTAGGC |
| mHES7 | CGGGAGCGAGCTGAGAATAG | CACGGCGAACTCCAGTATCT |
| mTBP | CCCCACAACTCTTCCATTCT | GCAGGAGTGATAGGGGTCAT |
| huTBP | TGCACAGGAGCCAAGAGTGAA | CACATCACAGCTCCCCACCA |

Table S3. The antibody, inhibitor and antagonist information used in this study

| For flow cytometry staining | | | |
| --- | --- | --- | --- |
| Antibody | Dilution | Catalog | Manufacturer |
| Anti-mouse CD16/32 | 1:100 | 101302 | BioLegend |
| Anti-mouse CD45 | 1:100 | 103108 | BioLegend |
| Anti-mouse CD45.2 | 1:100 | 109824 | BioLegend |
| Anti-mouse CD45R/B220 | 1:80 | 103207 | BioLegend |
| Anti-mouse CD11b | 1:80 | 101208 | BioLegend |
|  | 1:80 | 101243 | BioLegend |
| Anti-mouse Gr-1 | 1:80 | 108411 | BioLegend |
| Anti-mouse Ly6C | 1:100 | 128012 | BioLegend |
|  | 1:20 | 128033 | BioLegend |
| Anti-mouse Ly6G | 1:100 | 127624 | BioLegend |
| Anti-mouse F4/80 | 1:80 | 123116 | BioLegend |
| Anti-mouse MHCⅡ | 1:80 | 107628 | BioLegend |
| Anti-mouse CD206 | 1:80 | 141716 | BioLegend |
| Anti-mouse CD3e | 1:80 | 553061 | BD Biosciences |
| Anti-mouse CD3 | 1:80 | 100205 | BioLegend |
| Anti-mouse CD3ε | 1:100 | 155604 | BioLegend |
| Anti-mouse CD8a | 1:80 | 100706 | BioLegend |
|  | 1:80 | 100707 | BioLegend |
| Anti-mouse IFNγ | 1:50 | 505850 | BioLegend |
| Anti-mouse I-A/I-E | 1:200 | 107622 | BioLegend |
| Anti-mouse CD11c | 1:300 | 117349 | BioLegend |
| Anti-mouse NKp46 | 1:20 | 137608 | BioLegend |
| Anti-mouse CD4 | 1:80 | 100511 | BioLegend |
| Anti-mouse CD19 | 1:80 | 115508 | BioLegend |
| Anti-mouse CD127 | 1:20 | 135009 | BioLegend |
| Anti-mouse CD117 (c-Kit) | 1:50 | 105805 | BioLegend |
| Anti-mouse Ly-6A/E (Sca-1) | 1:80 | 108111 | BioLegend |
| Anti-mouse CD34 | 1:40 | 152209 | BioLegend |
| Anti-mouse FcγR | 1:80 | 101323 | BioLegend |
| Anti-mouse CD115 | 1:100 | 135523 | BioLegend |
| Anti-mouse Ly6C | 1:20 | 128033 | BioLegend |
| Anti-mouse CD31 | 1:100 | 553373 | BD Biosciences |
| Anti-mouse CD140b | 1:100 | 136006 | BioLegend |
| Anti-mouse CD24 | 1:40 | 138506 | BioLegend |
| Anti-mouse CD29 | 1:80 | 102226 | BioLegend |
| For western blotting | | | |
| Antibody | Dilution | Catalog | Manufacturer |
| CCR6 | 1:1000 | NBP2-25220 | Novus |
| CXCR2 | 1:1000 | 217314 | Abcam |
| NOTCH1 | 1:1000 | 3608 | Cell signaling technology |
| NOTCH2 | 1:1000 | 5732 | Cell signaling technology |
| NOTCH3 | 1:1000 | 23426 | Abcam |
| HEY1 | 1:1000 | 19929-1-AP | Proteintech |
| ALDH1A1 | 1:1000 | A0157 | ABclonal |
| TUBULIN | 1:1000 | HC101-01 | TransGen |
| HRP-conjugated anti-Rabbit IgG | 1:5000 | HS101-01 | TransGen |
| HRP-conjugated anti-Mouse IgG | 1:5000 | HS201-01 | TransGen |
| For immunofluorescence staining | | | |
| Antibody | Dilution | Catalog | Manufacturer |
| Anti-mouse CD11b | 1:100 | 17800 | Cell signaling technology |
| Anti-mouse Ly6G | 1:200 | 88876 | Cell signaling technology |
| For immunohistochemistry staining | | | |
| Antibody | Dilution | Catalog | Manufacturer |
| Anti-human CCL20 | 1:100 | 9829 | Abcam |
| Anti-human CD33 | 1:200 | 269456 | Abcam |
| Anti-human CD15 | 1:100 | 4744 | Cell signaling technology |
| Anti-human ALDH1A1 | 1:100 | 52492 | Abcam |
| For neutralization experiment | | | |
| Antibody | Catalog | | Manufacturer |
| Anti-lgG | MAB0061 | | R&D Systems |
| Anti-CXCL2 | MAB452-100 | | R&D Systems |
| For inhibition experiment | | | |
| Inhibitor or antagonist | Catalog | | Manufacturer |
| RO4929097 | HY-11102 | | MCE |
| SB225002 | S7651 | | Selleck |

Other Supplementary Materials for this manuscript:


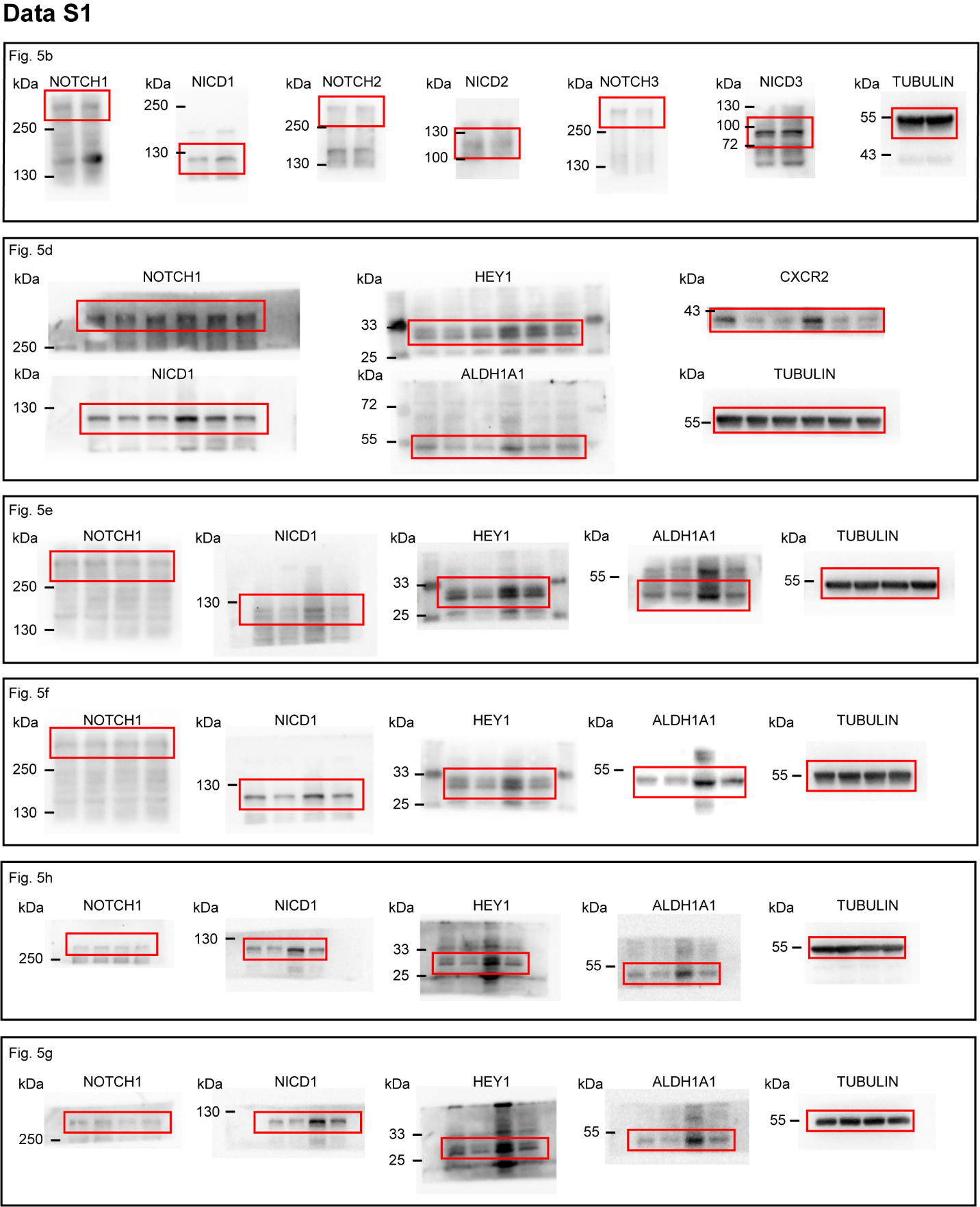


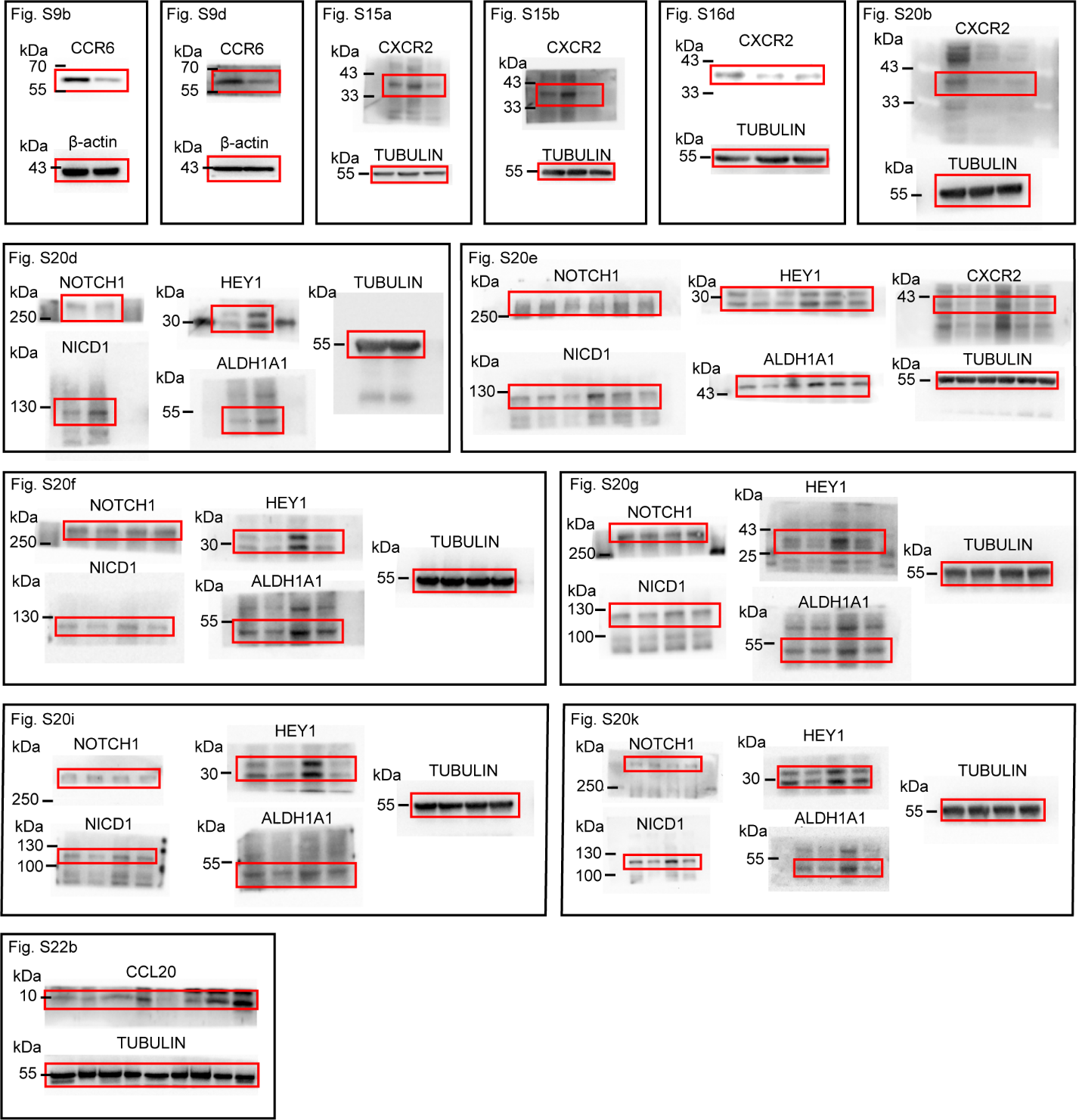


Data S1. The raw data of Western blots

Original and uncropped images of Western blots.


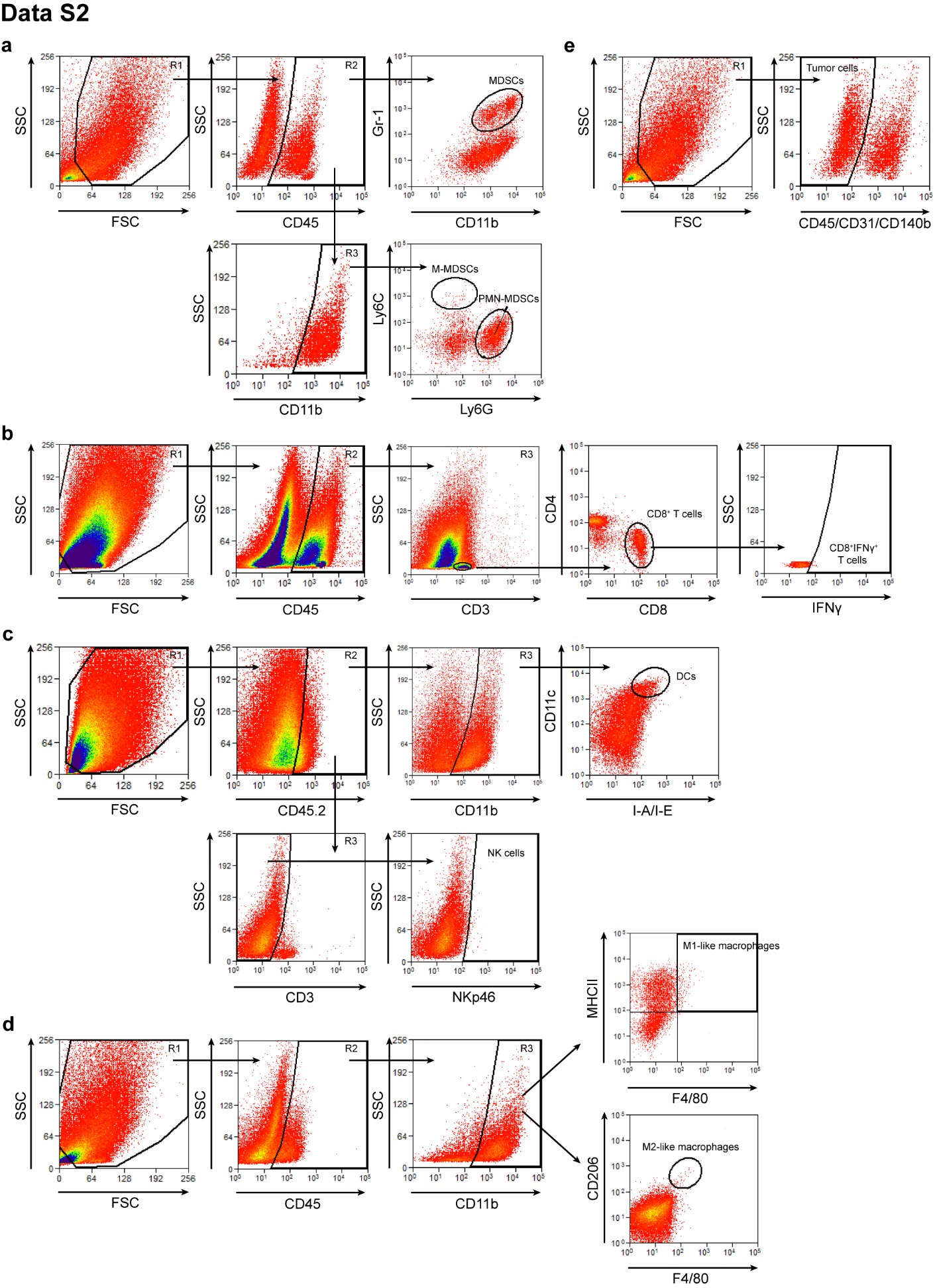


Data S2. Gating strategies for flow cytometries

**a-e** Gating strategies for the flow cytometries in Fig. 1f and 1i-k (a), Fig. 1g and 1h (b), Fig. S4d and S4e (c), Fig. S4a-c (d) and Fig. 4f (e).
